# ACSL3 inhibits ferroptosis in gastric cancer through the activation of unfolded protein response following endoplasmic reticulum stress

**DOI:** 10.1038/s41419-026-09068-3

**Published:** 2026-07-07

**Authors:** Honglei Wang, Jie Jiao, Wenshuo Yang, Danping Sun, Meng Wei, Guorui Sun, Zhibo Yan, Yize Liang, Zewei Cheng, Yongqi Yan, Menghui Wang, Yangjia Li, Xiaohan Cui, Wenbin Yu

**Affiliations:** Department of Gastrointestinal Surgery, General Surgery, Qilu Hospital of Shandong University, Jinan, China

**Keywords:** Gastric cancer, Cell death, Proteolysis

## Abstract

The long-chain acyl-CoA synthetase (ACSL) family has been associated with tumor progression across various cancer types. However, the function of the ACSL family in gastric cancer (GC) remains poorly understood. Comprehensive investigations employing in vivo and in vitro experiments demonstrate that ACSL3 suppresses ferroptosis and drives GC progression. Mechanistically, ACSL3 facilitated YY1 nuclear translocation, triggering endoplasmic reticulum (ER) stress and subsequent activation of the unfolded protein response (UPR). Genome-wide binding analysis revealed that YY1 directly binds to the USP37 promoter, enhancing its transcriptional activation. Furthermore, a novel interaction was identified between USP37 and PERK, a pivotal UPR regulator, wherein USP37 mediates K29-linked deubiquitination of PERK. PERK stabilization upregulated SLC7A11 expression, thereby inhibiting ferroptosis and promoting tumor progression. Collectively, the findings establish a molecular cascade wherein ACSL3 mediates ER stress-mediated UPR activation through the YY1/USP37/PERK axis, suppressing ferroptosis and accelerating GC progression, identifying ACSL3 as a potential therapeutic target for GC treatment.

## Introduction

GC ranks as the fifth most prevalent cancer globally [1]. The study has shown that by 2040, the annual new cases of GC in the world will reach 1.77 million [2]. Although the investigators have studied the biological features of GC extensively, surgery or endoscopic resection is still the main modality to treat GC [3]. There is still a considerable proportion of GC patients with postoperative recurrence, leading to poor prognosis [4]. Therefore, there is still an urgent need to find new diagnostic and therapeutic targets for GC.

To sustain their rapid proliferation, progression, and immune evasion capabilities, tumor cells undergo substantial alterations in their metabolic pathways and energy utilization [5]. Fatty acids (FAs), as elemental constituents of the lipidome, are multifunctional in the context of tumor biology. They are indispensable not just as essential energy reservoirs, but also as critical architectural components of cellular membrane architecture. Moreover, FAs are pivotal in modulating various oncogenic signaling cascades, where they act as key signaling entities integral to the pathogenesis and progression of cancer [6]. Notably, emerging evidence has established a strong correlation between FA metabolism and critical cellular processes in tumorigenesis, including the regulation of ferroptosis, apoptosis, and the development of chemoresistance [7]. Particularly in GC, dysregulated FA metabolism has been demonstrated to provide substantial bioenergetic support for tumor initiation and progression while facilitating metastatic dissemination [8–10]. These studies have revealed the therapeutic potential of targeting FA metabolic pathways, suggesting that the profound understanding of the metabolic reprogramming in GC cells may yield novel strategies for prevention and treatment of this malignancy.

The ACSL family is a key enzyme that catalyzes a variety of FAs for subsequent metabolism, and its expression level has been shown to be correlated with the survival rate of patients with a variety of tumor types [11]. The ACSL family consists of five members with different intracellular localization and physiological roles. ACSL3 predominantly localizes to the lipid droplets, mitochondria, and ER within cellular compartments, where it assumes a critical role in the orchestration of lipid metabolism. Specifically, ACSL3 is essential for the regulation of lipid trafficking and the facilitation of FA uptake, thereby influencing cellular energetics and lipid homeostasis [12]. Current research indicates that ACSL3 expression is typically elevated across diverse types of cancer, and it influences the occurrence and progression of tumors via multiple mechanisms. In pancreatic cancer, ACSL3 up-regulated PAI-1 to increase the degree of tumor fibrosis and the number of immunosuppressive cells, and facilitated the progression of pancreatic cancer [13]. In breast cancer, ACSL3 regulates breast cancer progression through reprogramming of lipid metabolism[14]. In addition, ACSL3 promotes prostate cancer progression by regulating intratumoral steroid synthesis and androgen metabolism [15]. In summary, ACSL3 may be used as a prognostic indicator and a therapeutic target for cancer. Understanding the regulatory mechanism of ACSL3 will help to develop a reasonable tumor treatment and therapeutic strategies. However, the precise function of ACSL3 in GC is still lack of in-depth study.

This study elucidated the molecular mechanisms underlying ACSL3-mediated ferroptosis suppression in GC cells. Our findings revealed a previously unrecognized regulatory axis, ACSL3-YY1-USP37-PERK, through which ACSL3 orchestrated GC progression. This novel mechanistic finding not only advanced the understanding of ferroptosis regulation in GC but also established ACSL3 as a potential indicator and a therapeutic target for clinical treatment and diagnosis strategies in GC.

## Materials and methods

### Patient samples

The GC tissues, and normal gastric tissue and basic clinical information were collected from 360 patients who underwent radical GC surgery at the Department of Gastrointestinal Surgery, Qilu Hospital of Shandong University, from January 2018 to December 2019. All patients who participated in the study signed an informed consent form in advance, and the data used were approved in advance by the Research Ethics Committee of Qilu Hospital of Shandong University and complied with the ethical guidelines of the World Medical Association Declaration of Helsinki. Relevant patient characteristics are detailed in Suppl. Table 1.

### Cell culture and cell lines

The following human GC cell lines were used in this study: HGC-27, MKN45, BGC-823, AGS, and Ncl-N87. HGC-27 was established from a lymph node metastatic lesion of a Japanese patient with undifferentiated GC. MKN45 was established from a liver metastasis of a 35-year-old female patient with poorly differentiated gastric adenocarcinoma. BGC-823 was derived from the primary GC tissue of a male patient with poorly differentiated gastric adenocarcinoma. AGS was established from a surgically resected tumor of a 54-year-old Caucasian female patient with gastric adenocarcinoma who had received no prior anticancer therapy. Ncl-N87 was established from a liver metastatic lesion of a male patient with GC. The human normal gastric mucosal epithelial cell line GES-1 was used as a non-cancerous control. All cell lines were obtained from Fenghui Biotechnology (Hunan, China). The HGC-27, MKN45, BGC-823, Ncl-N87 and GES-1 cells were cultured in medium consisting of 10% fetal bovine serum (26010074, Gibco, Paisley, UK), 1640 medium (C3010-0500, Vivacell, Shanghai, China) and 1% penicillin/streptomycin antibiotics (P1400, Solarbio, Beijing, China) in air conditions of 37 °C and 5% CO2. The AGS cells were cultured in a medium consisting of 12% fetal bovine serum (26010074, Gibco), DMEM/F12 medium (C3132-0500, Vivacell), and 1% penicillin/ streptomycin antibiotics (P1400, Solarbio). All cells were passaged no more than 20 times. All cell lines utilized in this study were authenticated by short tandem repeat and confirmed to be free of mycoplasma contamination.

### Small interfering RNA (siRNA) transfection and plasmid transfection

GC cells were evenly seeded into a six-well plate, allowed to adhere stably to the plate surface, and reach a confluence of approximately 70% before siRNA was transfected with iMax (13778150, Invitrogen, California, USA). The plasmids were transfected using P3000 (L3000075, Invitrogen) and Lipofectamine 3000 (L3000075, Invitrogen) transfection reagents. RNA was extracted after 24 h of transfection, and protein was extracted after 48 h of transfection.

### Establishment of stable expression cell lines

psPAX2 and pMD2.G and lentiviral vectors were used to co-transfect into HEK-293T cells, and the supernatant was collected after 48 h, which was used to infect the GC cells for 24 h with puromycin (P1400, Solarbio). Stable cell lines were screened, and then knockdown and overexpression of relevant genes were verified at the mRNA and protein levels using RT-qPCR and western blot techniques. The siRNA and shRNA sequences are shown in Suppl. Table 2.

### RNA extraction and RT-qPCR analysis

Utilizing the RNA extraction kit (220011, Fastagen, Shanghai, China), total RNA was isolated from the pretreated GC cells or tissues, the concentration was measured. Reverse transcription to cDNA was performed by the HiScript III RT SuperMix for qPCR (R323-01, Vazyme, Nanjing, China), and amplification was performed. Primer sequences were obtained from Biosune Biotechnology Ltd (Shanghai, China). The internal reference gene was GAPDH, and the relative RNA expression levels were quantified by the 2^-ΔΔCt^ method. Relevant primers are detailed in Suppl. Table 3.

### Western blot analysis

GC cell proteins were isolated using a RIPA lysate (P0013B, Beyotime Biotechnology, Shanghai, China) with the addition of 1% phenylmethanesulfonyl fluoride (P0100, Solarbio) and 1% phosphatase inhibitor (P1260, Solarbio) and then the proteins were resolved in 10% sodium dodecyl sulfate-polyacrylamide gel electrophoresis and subsequently transferred to the polyvinylidene fluoride (PVDF) membrane (ISEQ00010, Millipore, Massachusetts, USA). The PVDF membrane was closed with 5% skimmed milk for 1.5 h at room temperature before incubating the membranes with specific primary antibodies at 4 °C overnight. The PVDF membrane was incubated with secondary antibodies of goat anti-mouse antibody (1:10000, ZB-2305, ZSGB-BIO, Beijing, China) or goat anti-rabbit antibody (1:10000, ZB-2301, ZSGB-BIO) for 2 h at room temperature and then visualized using ECL luminescent liquid (WBKLS0500, Millipore). The relevant antibodies are detailed in Suppl. Table 4.

### In vivo tumorigenesis

Four-week-old BALB/c nude mice were procured from Gempharmatech Co., Ltd, Jiangsu, China. Stable cell lines constructed by lentivirus were injected subcutaneously in the left axilla of nude mice, and each nude mouse was inoculated with 1 × 10^7^ cells (*n* = 8, random allocation). Tumor size was recorded by vernier caliper and tumor volume was calculated every 4 days after inoculation, and the tumors were collected and weighed on the 16th day, and the tumor slices were sliced and then evaluated for the expression level of the relevant protein by immunohistochemistry (IHC) staining.

### IHC staining

The animal tissues and human GC tissues were preserved using 4% paraformaldehyde. The tissue was then embedded and fixed in paraffin and sectioned. Tissues were then deparaffinized, antigenically repaired and blocked, and incubated overnight at 4 °C with the specific primary antibody, and the secondary antibody incubated at room temperature and then stained using diaminobenzidine, while the nuclei of the cells were stained with hematoxylin (M&R HRP/DAB Detection IHC Kit, HC301-01, Vazyme). Scoring was performed by the H-score method according to the percentage of positively stained GC cells and the intensity of staining, and the scores were plotted on a histogram.

### LPO detection

Stabilized adherent GC cells were washed with HBSS for one time, then 10 µmmol/L liperfluo working solution (LPO assay kit, L248, Dojindo, Beijing, China) was added and incubated for 30 min at 37 °C. The cells were then digested with trypsin and collected, then washed twice with HBSS and resuspended. The LPO level was determined by flow cytometry at 488 nm.

### Intracellular Fe^2+^ detection

Twelve-well plates with adherent GC cells were incubated with 1 mmol/L FerroOrange solution (the FerroOrange assay kit, F374, Dojindo) for 30 min at 37 °C, and then the Fe^2+^ levels were observed under a fluorescence microscope.

### MDA detection

The samples added to the MDA assay workup assay kit (S0131M, Beyotime), and the samples were then incubated at 100 °C for 15 min to facilitate the reaction, followed by cooling to room temperature. After reaching room temperature, the samples were subjected to centrifugation at 1000 × *g* for 10 min to separate the supernatant containing the MDA-reactive substances for further analysis. A volume of 200 μL of the supernatant was carefully transferred into a 96-well plate. The absorbance of the samples was measured at 532 nm, and the MDA content was calculated.

### ROS detection

Add an appropriate amount of 1:1000 diluted DCFH-DA stock solution (the ROS assay kit, S0033S, Beyotime) to the twelve-well plate with adherent GC cells and incubate at 37 °C for 30 min, avoiding light, wash with an appropriate amount of HBSS 3 times, and then observe the ROS level under a fluorescence microscope.

### TEM assay

Appropriate amount of electron microscope fixative was added to the 6 well plate with adherent GC cells, and the cells were fixed for 5 min at room temperature and protected from light and then collected in an EP tube, centrifuged at 1000 rpm for 5 min, and the supernatant was discarded, and the GC cells were resuspended with a new electron microscope fixative and fixed for 30 min at room temperature and protected from light. Servicebio Co., Ltd. (Wuhan, China) performed the standard protocols.

### Cut & RUN assay

Experiments were performed using 5 × 10^5^ GC cells per sample using the Hyperactive Universal Cut & RUN Assay Kit for Illumina (TD904, Vazyme). The resulting yield was purified by 13 cycles of PCR amplification and sequenced using the Illumina platform.

### Dual-luciferase assay

The binding site of USP37 promoter region fragments (5’-GCCATC-3’) was constructed into the pGL3-basic vector, and YY1 was erected into the pcDNA3.1–3x flag vector. The firefly luciferase plasmids YY1 (WT and mut) and YY1 expression vectors (containing vector or oe-YY1), and the pRL-TK Renilla luciferase vector (internal reference) into HEK-293T cells. The pRL-TK vector, encoding Renilla luciferase as an internal control, was also transfected into HEK-293T cells. After 48 h, the Dual-Luciferase Reporter Assay Kits (11402ES60, Yeasen, Shanghai, China) were employed to quantify the activities of both firefly and Renilla luciferase activities.

### Co-IP assay

Add IP lysis buffer (P2179S, Beyotime) to the pretreated GC cells. The cell lysis process was performed on ice for 30 minutes and then centrifuged for 5 min at 14,000 × *g* and 4 °C to collect the supernatant. The supernatant was mixed with the corresponding specific primary antibody and incubated overnight at 4 °C. Sepharose-conjugated Protein A + G beads (sc-2003, Santa Cruz, California, USA) were added and incubated at room temperature for 4 h. The bead-antibody complex was then centrifuged to pellet the beads, which were washed meticulously, and then boiled and heated to elute the bound proteins for 10 minutes for subsequent experiments.

### Statistical analysis

SPSS software version 17.0 and Graphpad Prism 7.0 software were used for statistical analysis. All data were analyzed using Student’s t-test or one way ANOVA method. Bars and errors represent the mean ± standard deviation of repeated measurements, *p* < 0.05 was considered significant. Three independent replications of the experiments were performed, except for the clinical samples and in vivo experiments.

## Results

### High expression of ACSL3 in GC tissues is associated with adverse prognostic outcomes in patients with GC

**Fig. 1:**
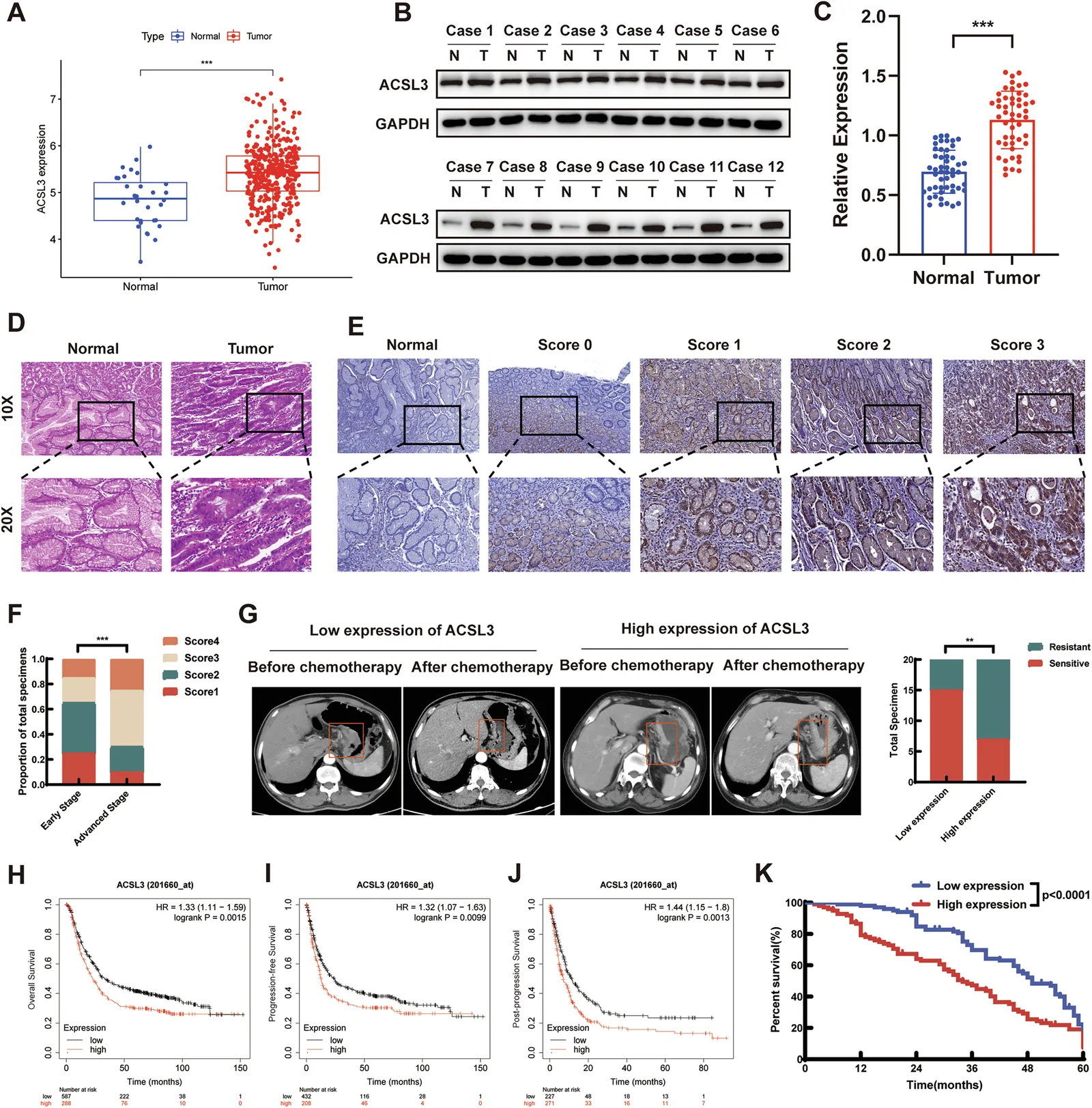
High expression of ACSL3 in GC tissues was associated with adverse prognostic outcomes in patients with GC. **A** Analysis of the TCGA dataset showed that ACSL3 was significantly up-regulated in GC tissues. **B** Compared with normal gastric mucosal tissues, ACSL3 was significantly highly expressed in GC tissues. **C** RT-qPCR results showed that ACSL3 mRNA expression in GC tissues was significantly higher than that in normal gastric mucosal tissues. **D** Hematoxylin and eosin stains of GC tissue and normal gastric tissue. **E** The expression of ACSL3 in different T-stage GC tissues and normal gastric tissues was detected by immunohistochemical staining. **F** Evaluation of ACSL3 expression in early GC tissues and progressive gastric tissues using the H-score method. **G** CT images of GC patients receiving the same cycle of L-OHP plus S-1 (Tegafur/ Gimeracil/ Oteracil Potassium) regimen (SOX regimen) showed that patients with high ACSL3 expression are less sensitive to chemotherapy. Statistical analysis was performed using the chi-square test (χ² = 7.38, *p* = 0.007). **H–J** Aplan-Meier Plotter analysis showed that high expression of ACSL3 led to poor prognosis in GC patients. **K** Overall survival follow-up of GC patients showed that high expression of ACSL3 leads to poor prognosis in GC patients. Data are represented as the mean ± SD. Statistical analysis was performed using Student’s t-test and survival analysis employed the Log-rank (Mantel-Cox) test, *p < 0.05; **p < 0.01; ***p < 0.001.

Previous studies have established that elevated levels of monounsaturated fatty acids (MUFAs) lead to a reduction in ferroptosis sensitivity, promote tumor progression, and correlate with poor clinical outcomes [16, 17]. Under treatment with (a) PBS control, (b) oxaliplatin treatment (L-OHP, 5 mg/kg, i.p.), (c) oleic acid administration (OA, 2 mg/kg, intratumoral), and (d) OA plus L-OHP combination, the results demonstrated that OA significantly enhanced tumor progression and reduced L-OHP sensitivity in GC (Fig. S1A–C). Given that ACSL3 serves as the key regulatory enzyme in MUFA metabolism and mediates MUFA-dependent ferroptosis suppression [17]. Analysis by TCGA dataset revealed that ACSL3 was significantly upregulated in GC tissues compared to adjacent normal tissues (Fig. 1A). To validate these findings, 50 paired GC and adjacent normal tissues from GC patients were examined, revealing significant ACSL3 upregulation at mRNA and protein levels in tumor tissues (Fig. 1B, C). Histopathological evaluation through hematoxylin-eosin (HE) and IHC staining analysis across different T-stages demonstrated progressively increased ACSL3 expression in advanced-stage GC compared to early-stage GC (Fig. 1D–F). Clinical correlation analysis of preoperative imaging data from neoadjuvant chemotherapy patients revealed that high ACSL3 expression was associated with chemotherapy resistance (Fig. 1G). Kaplan-Meier analysis of TCGA data demonstrated that elevated ACSL3 expression significantly reduced overall survival, progression-free survival, and post-progression survival in GC patients (Fig. 1H–J). To further validate these findings, tumor samples from 200 GC patients (100 high and 100 low ACSL3 expression tissues) with 5-year follow-up data were analyzed, confirming that high ACSL3 expression was significantly associated with poorer prognosis (Fig. 1K). Taken together, these results indicated that ACSL3 was highly expressed in GC tissues and correlated with poor prognosis of GC patients.

### ACSL3 inhibited ferroptosis in GC cells

**Fig. 2:**
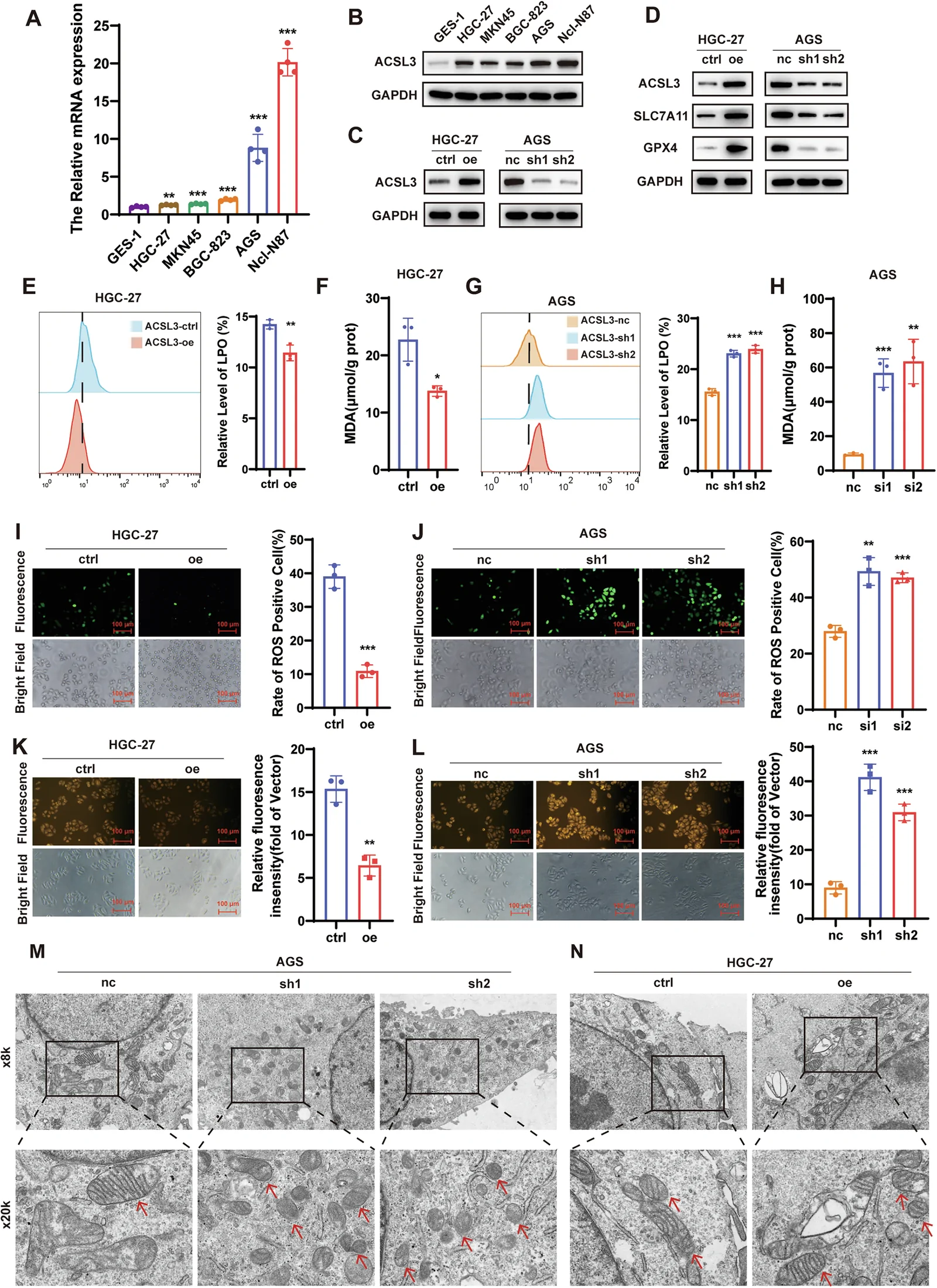
ACSL3 inhibited ferroptosis in GC cells. **A** Compared with gastric mucosal epithelial cell GES-1, ACSL3 was highly expressed at the mRNA level in GC cells HGC-27, MKN45, BGC-823, AGS and Ncl-N87. **B** The result of western blot showed ACSL3 was also highly expressed at the protein level in GC cells HGC-27, MKN45, BGC-823, AGS and Ncl-N87. **C** Western blot assay of ACSL3 expression in HGC-27 transfected with ACSL3 or vector control and in AGS transfected with shACSL3 RNA or shRNA control. **D** Western blot detection of ACSL3, SLC7A11 and GPX4 expression levels in the groups of GC cells, ACSL3-ctrl, ACSL3-oe, ACSL3-nc, ACSL3-sh1, ACSL3-sh2. **E** Cell flow assay detection of lipid peroxides levels in the groups of ACSL3-ctrl, ACSL3-oe in HGC-27. **F** Detection of MDA levels in the groups of ACSL3-ctrl, ACSL3-oe in HGC-27. **G** Cell flow assay detection of lipid peroxides levels in the groups of ACSL3-nc, ACSL3-sh1, ACSL3-sh2 in AGS. **H** Detection of MDA levels in the groups of ACSL3-nc, ACSL3-sh1, ACSL3-sh2 in AGS. **I** Detection of ROS levels in the groups of ACSL3-ctrl, ACSL3-oe in HGC-27. **J** Detection of ROS levels in the groups of ACSL3-nc, ACSL3-sh1, ACSL3-sh2 in AGS. **K** Detection of Fe^2+^ levels in the groups of ACSL3-ctrl, ACSL3-oe in HGC-27. **L** Detection of Fe^2+^ levels in the groups of ACSL3-nc, ACSL3-sh1, ACSL3-sh2 in AGS. **M** Morphology of mitochondria in the groups of ACSL3-nc, ACSL3-sh1, ACSL3-sh2 in AGS, observed by electron microscopy. **N** Morphology of mitochondria in the groups of ACSL3-ctrl, ACSL3-oe in HGC-27, observed by electron microscopy. Data are represented as the mean ± SD. Statistical analysis was performed using Student’s t-test, *p < 0.05; **p < 0.01; ***p < 0.001.

Previous studies have shown that ferroptosis, a distinct type of cell death characterized by LPO and iron overload, was an important process in the progression of GC. Moreover, ACSL3 functioned as an inhibitor of ferroptosis in tumor cells. To verify whether ACSL3 functioned to inhibit ferroptosis in GC cells, it was found that the expression of ACSL3 was significantly elevated at mRNA and protein levels in the GC cell lines by comparing the differences in ACSL3 expression between GES-1 and GC cell lines HGC-27, MKN45, BGC-823, AGS, Ncl-N87 (Fig. 2A, B). Subsequently, stable knockdown or overexpression GC cell lines were constructed for studying the role of ACSL3 in GC development (Fig. S1D; Fig. 2C). Existing studies have shown that ACSL3 plays a role as a key enzyme involved in FA oxidation and maintenance of lipid droplet formation to reduce the level of intracellular ferroptosis [17, 18]. Western blot analysis revealed that knockdown of ACSL3 decreased the expression levels of SLC7A11 and GPX4 in AGS and Ncl-N87, whereas overexpression of ACSL3 increased their expression in HGC-27 and MKN45 (Fig. S1E; Fig. 2D). The results showed that overexpression of ACSL3 decreased LPO and MDA levels in HGC-27 (Fig. 2E, F) and MKN45 (Fig S1F, G), and knockdown of ACSL3 increased LPO and MDA levels in AGS (Fig. 2G, H) and Ncl-N87 (Fig. S1H, I). Overexpression of ACSL3 decreased these levels of Fe^2+^ and ROS in HGC-27 (Fig. 2I, K) and MKN45 (Fig. S1J, L), while knockdown of ACSL3 increased them in AGS (Fig. 2J, L) and Ncl-N87 (Fig. S1K, M). Furthermore, TEM analysis revealed that knockdown of ACSL3 in AGS markedly reduces mitochondrial volume, causes a reduction or loss of mitochondrial cristae, increases mitochondrial membrane density, and induces rupture of the outer mitochondrial membrane (Fig. 2M). Conversely, ACSL3 overexpression in HGC-27 alleviated these changes. These ultrastructural alterations—namely mitochondrial shrinkage and cristae loss—are characteristic features of ferroptosis, distinguishing it from apoptosis, which typically preserves mitochondrial structure, and from necrosis, which is associated with mitochondrial swelling. And overexpression of ACSL3 significantly reduced the characteristic ferroptosis-associated changes in the mitochondrial morphology of HGC-27 cells (Fig. 2N). These results suggested that ACSL3 could inhibits ferroptosis in GC cells. Subsequently, ACSL3‑knockdown GC cells were treated with the classical ferroptosis inhibitor ferrostatin‑1 (2 μM) for 12 h to further demonstrate that the cell death phenotype regulated by ACSL3 is specifically dependent on ferroptosis, rather than general oxidative stress or alternative cell death pathways. The results showed that the elevated levels of ROS (Fig. S2A), Fe²⁺ (Fig. S2B), LPO (Fig. S2E), and MDA (Fig. S2F) induced by ACSL3 knockdown in AGS were counteracted by ferrostatin-1 treatment, and similar results were observed in Ncl-N87 (Fig. S2C, D, G, H). Subsequently, ACSL3-knockdown GC cells were treated with the classical ferroptosis inducer Erastin (5 μM) for 12 h to evaluate changes in the sensitivity of GC cells to ferroptosis. The results showed that ACSL3 knockdown combined with Erastin treatment significantly elevated the levels of ROS (Fig. S3A), Fe²⁺ (Fig. S3B), LPO (Fig. S3E), and MDA (Fig. S3F) in AGS, and similar results were observed in NCI-N87 (Fig. S3C, D, G, H). These findings indicate that ACSL3 inhibition significantly enhances the sensitivity of GC cells to ferroptosis. ACSL3 overexpression reduced the elevations of ROS (Fig. S4A), Fe²⁺ (Fig. S4B), LPO (Fig. S4E), and MDA (Fig. S4F) induced by Erastin in HGC-27, and similar results were observed in MKN-45 (Fig. S4C, D, G, H). These results demonstrate that ACSL3 knockdown enhances the sensitivity of GC cells to ferroptosis, whereas ACSL3 overexpression reduces it. Therefore, targeting ACSL3 to induce ferroptosis may represent an effective therapeutic strategy for GC.

### YY1 facilitated USP37 gene expression through promoter binding

**Fig. 3:**
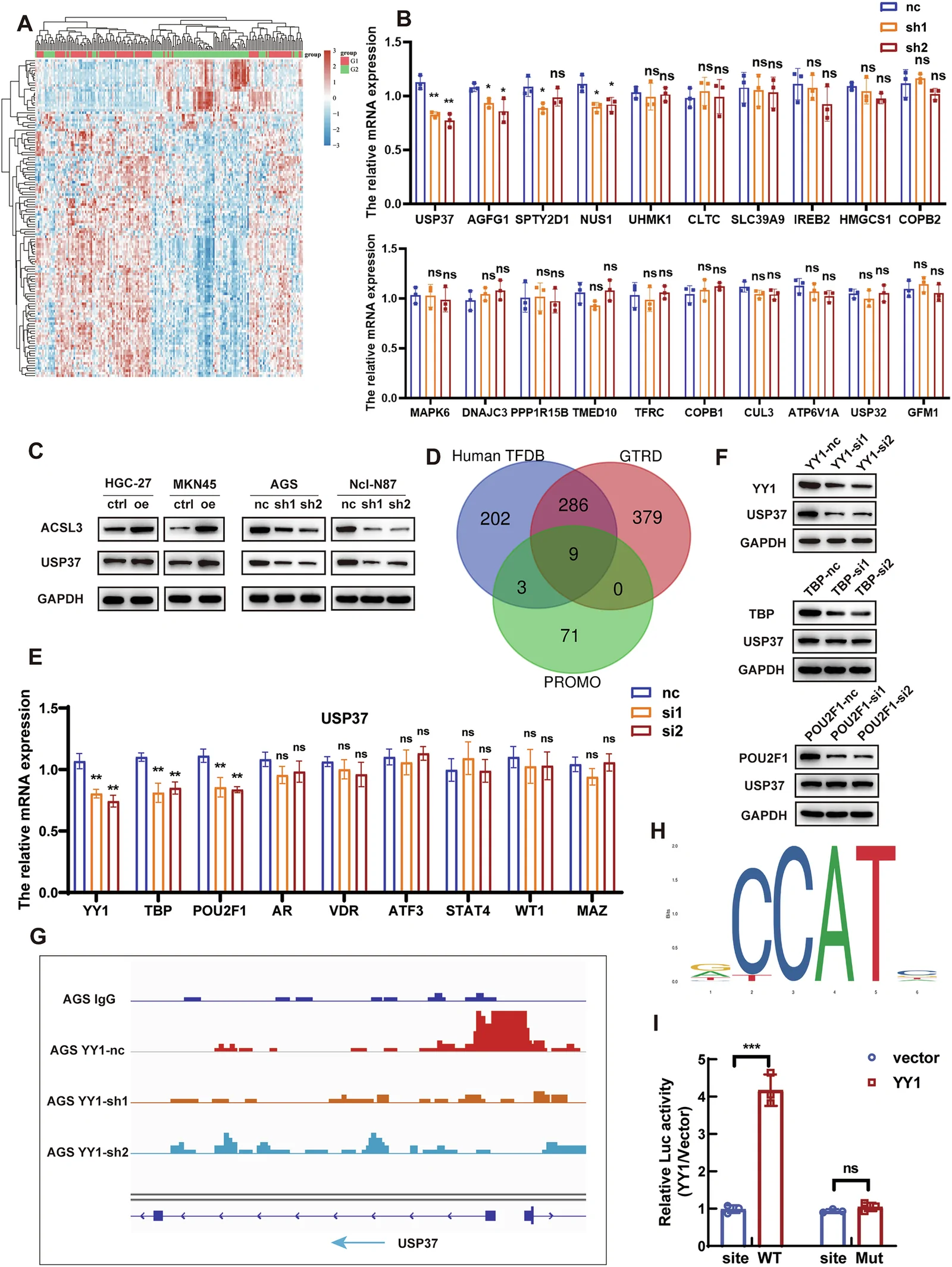
ACSL3-mediated regulation of YY1 nuclear translocation augments ER stress response and suppressed ferroptosis in GC cells. **A** Bioinformatics analysis based on TCGA database was performed to predict the downstream genes of ACSL3. G1: high expression of ACSL3, G2: low expression of ACSL3. **B** Analysis of mRNA expression changes in the top 20 most differentiated genes after verifying knockdown of ACSL3 in AGS. **C** Western blot detection of ACSL3, USP37 expression levels in the groups of GC cells, ACSL3-ctrl, ACSL3-oe, ACSL3-nc, ACSL3-sh1, ACSL3-sh2. **D** The USP37 TF prediction in Human TFDB, GTRD, and PROMO databases. **E** The USP37 mRNA levels were detected after knocking down each of these nine genes publicly owned by the 3 databases in AGS. **F** Western blot detection of YY1, TBP, POU2F1 and USP37 expression levels after knockdown of YY1, TBP and POU2F1 respectively in AGS. **G** Detection of YY1 binding abundance at USP37 promoter after knockdown of YY1 in AGS by Cut & RUN. **H** Motif Logo of the binding site sequence with the most pronounced reduction in binding abundance of YY1 at the USP37 promoter. **I** Dual-luciferase reporter assay showing luciferase activities of AGS cells transfected with Vector-oe and YY1-oe. Data are represented as the mean ± SD. Statistical analysis was performed using Student’s t-test, *p < 0.05; **p < 0.01; ***p < 0.001.

To further investigate the specific mechanism of ACSL3 inhibiting ferroptosis in GC, bioinformatics analysis based on TCGA database was performed to predict the downstream genes of ACSL3 (Fig. 3A). Following ACSL3 knockdown in AGS cells, the mRNA expression changes of the top 20 genes with the most significant alterations in the bioinformatic analysis were examined. USP37 exhibited the greatest fold change among these genes. Notably, other genes such as AGFG1 and NUS1 were also significantly dysregulated upon ACSL3 knockdown (Fig. 3B). USP37 encodes a deubiquitinating enzyme. Deubiquitinases have recently been shown to regulate ferroptosis by controlling the stability of GPX4 [19, 20]. In GC, PLAGL2 has been reported to promote the proliferation and migration of GC cells through USP37-mediated Snail1 deubiquitination [21]. In contrast, AGFG1, which is involved in vesicular transport, and NUS1, which is associated with congenital disorders of glycosylation and neurological diseases, have no known link to ferroptosis. Among the top 20 genes, USP37 exhibited the largest expression change and was therefore selected for functional validation. Subsequently, the results showed that knockdown of ACSL3 resulted in decreased expression levels of USP37 in AGS and Ncl-N87, and overexpression of ACSL3 resulted in increased expression levels of USP37 in HGC-27 and MKN45 (Fig. 3C). There is no evidence that USP37 expression is directly regulated by the ACSL family. Therefore, we hypothesized that ACSL3 may indirectly affect USP37 expression in some way. The intersection of USP37 transcription factor (TF) predictions from the Human TFDB, GTRD, and PROMO databases yielded 9 TFs (Fig. 3D), namely YY1, TBP, POU2F1, AR, VDR, ATF3, STAT4, WT1, and MAZ. These 9 TFs were knocked down in AGS, and it was found that the mRNA level of USP37 was significantly decreased after knocking down YY1, TBP, and POU2F1 (Fig. 3E). Only the knockdown of YY1 was observed to result in a significant decrease in USP37 protein level (Fig. 3F). Cut & RUN results showed that knockdown of YY1 in AGS significantly reduced YY1 binding abundance at the USP37 promoter, and the binding site sequence was 5’-GCCATC-3’ (Fig. 3G, H). Dual-luciferase reporter assays showed that YY1 enhanced the activity of the USP37 promoter, whereas this facilitation was counteracted when the YY1 binding site within the promoter region was mutated (Fig. 3I). The results suggested that YY1 served as a TF of USP37, thereby elevating its expression. Meanwhile, USP37 had been identified as a downstream target gene of ACSL3, and YY1 may play a key role in the ACSL3 regulatory network that regulated USP37 expression.

### ACSL3-mediated regulation of YY1 nuclear translocation augments ER stress response and suppressed ferroptosis in GC cells

**Fig. 4:**
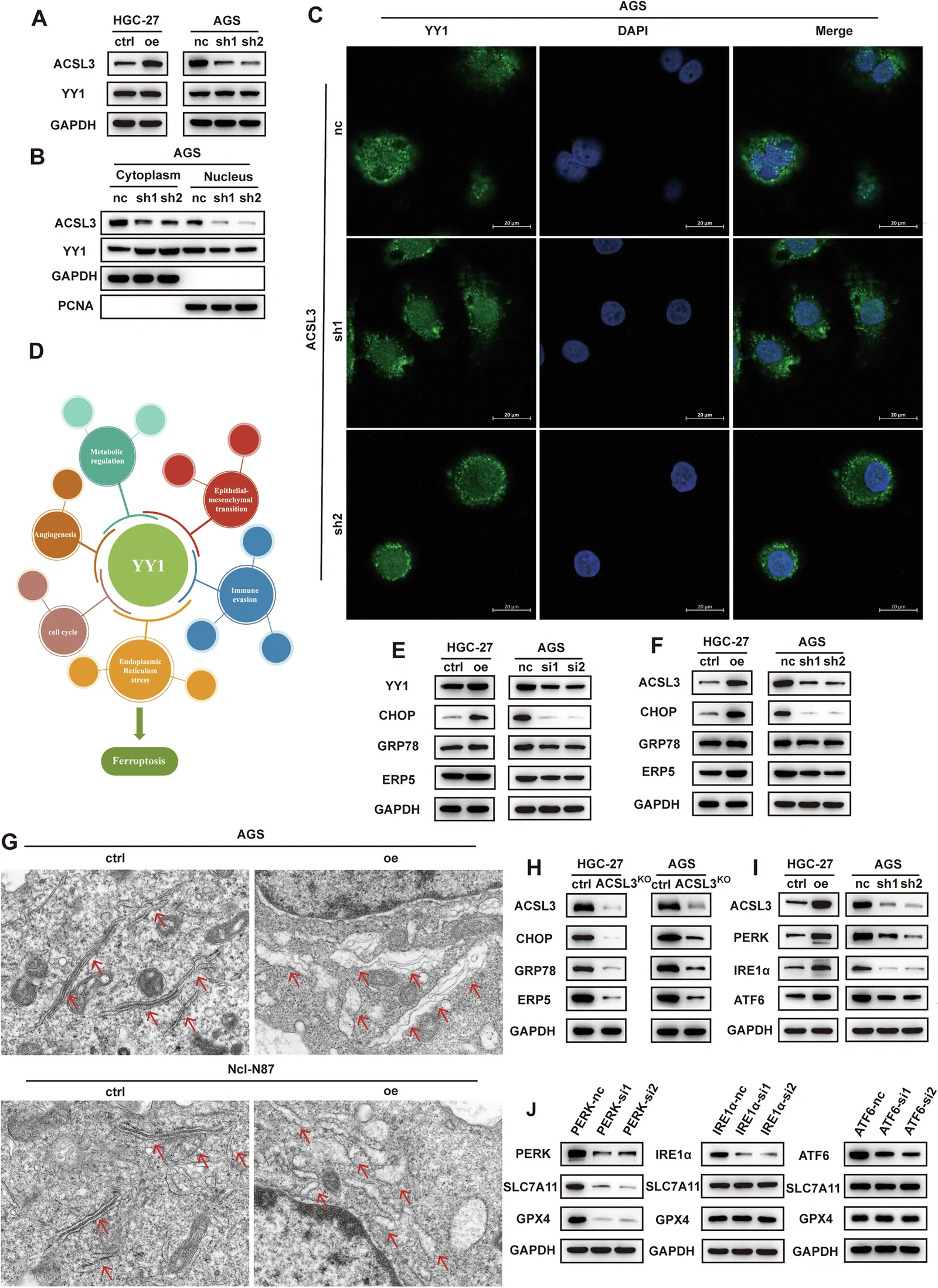
ACSL3 regulation of YY1 nuclear translocation promoted ER stress response and inhibited ferroptosis. **A** Western blot detection of ACSL3 and YY1 expression levels in the groups of GC cells, ACSL3-ctrl, ACSL3-oe, ACSL3-nc, ACSL3-sh1, ACSL3-sh2. **B** Western blot detection of ACSL3 and YY1 expression in the nucleus and the cytoplasm in AGS transfected with shACSL3 RNA or shRNA control. **C** Representative images of immunofluorescence staining on YY1 (green) and DAPI (blue) in AGS transfected with shACSL3 RNA or shRNA control. **D** Schematic representation of YY1 as a TF involved in a variety of biological processes. **E** Western blot detection of ER stress marker molecules in HGC-27 transfected with YY1 or vector control and in AGS transfected with siYY1 RNA or siRNA control. **F** Western blot detection of ER stress marker molecules in HGC-27 transfected with ACSL3 or vector control and in AGS transfected with shACSL3 RNA or shRNA control. **G** Morphology of ER in the groups of ACSL3-ctrl, ACSL3-oe in AGS and Ncl-N87, observed by electron microscopy. **H** Western blot detection of ER stress marker molecules in HGC-27 and AGS treated with control or CRISPR-mediated knockout of ACSL3 (KO). **I** Western blot detection of UPR marker molecules in HGC-27 transfected with ACSL3 or vector control and in AGS transfected with shACSL3 RNA or shRNA control. **J** Western blot detection of PERK, IRE1α, ATF6, SLC7A11 and GPX4 expression levels after knockdown of PERK, IRE1α and ATF6 respectively in AGS. Data are represented as the mean ± SD. Statistical analysis was performed using Student’s t-test, *p < 0.05; **p < 0.01; ***p < 0.001.

To further investigate the influence of ACSL3 on YY1 and explore the detailed regulatory mechanisms. Western blot analysis showed that neither ACSL3 knockdown nor overexpression altered YY1 protein levels (Fig. 4A; Fig. S5A). Studies have shown that YY1, as a TF, is involved in a variety of biological processes and that affecting its nuclear translocation could alter its TF function [22, 23]. Subsequently, knockdown of ACSL3 in AGS (Fig. 4B) and Ncl-N87 (Fig. S5B) resulted in a decrease in YY1 protein levels in the nucleus and an increase in YY1 protein levels in the cytoplasm. Immunolocalization results similarly showed that the knockdown of ACSL3 in AGS (Fig. 4C) and Ncl-N87 (Fig. S5C) promoted YY1 translocation from the nucleus to the cytoplasm. YY1 acts as a TF involved in a variety of biological processes, such as ER stress, cell cycle regulation, angiogenesis, metabolic regulation, epithelial-mesenchymal transition, and immune escape, among which ER stress is closely related to ferroptosis (Fig. 4D) [24, 25]. Subsequently, overexpression of YY1 was found to increase ER stress levels in HGC-27 (Fig. 4E) and MKN45 (Fig. S5D), whereas knockdown of YY1 showed the opposite result in AGS (Fig. 4E) and Ncl-N87 (Fig. S5D). Similarly, knockdown or overexpression of ACSL3 resulted in significant changes in ER stress levels (Fig. 4F; Fig. S5E). Examination of ER morphology by TEM in cell lines with stable YY1 expression alterations provided direct evidence that YY1 induces specific ER changes. The result revealed marked ER swelling, dilation, and vacuolation following ACSL3 overexpression, directly indicating elevated ER stress levels (Fig. 4G). Additionally, to address the question of whether ER stress might be epiphenomenal either due to forced protein expression or alterations in the lipidome, CRISPR-mediated knockout of ACSL3^KO^ resulted in a significant reduction in ER stress levels (Fig. 4H). Cells are able to sense protein misfolding or accumulation of unfolded proteins under ER stress and activates the UPR to alleviate ER stress through 3 pathways (including PERK, IRE1α, ATF6) [26]. To investigate the relationship between ACSL3 and UPR after ER stress, expression of marker proteins of the UPR was examined following ACSL3 overexpression or knockdown, and results showed that overexpression of ACSL3 increased the expression level of UPR marker molecules in HGC-27 (Fig. 4I) and MKN45 (Fig. S5F), whereas knockdown of ACSL3 showed the opposite result in AGS (Fig. 4I) and Ncl-N87 (Fig. S5F). Knockdown of three key genes of the UPR pathway revealed that knockdown of PERK in AGS resulted in a significant decrease in the protein expression levels of SLC7A11 and GPX4, whereas knockdown of IRE1α and ATF6 did not affect gene expression (Fig. 4J). And it has been shown that in GC, the PERK-EIF2α-ATF4-SLC7A11 pathway regulates ferroptosis in GC and significantly affects GC progression [23, 27]. We hypothesized that ACSL3 promotes ER stress and the PERK pathway in the UPR via YY1 modulation, consequently enhancing ferroptosis. To test the above hypothesis, the ER stress level, the UPR level, and the ferroptosis level were examined. And the results showed that knockdown of ACSL3 resulted in decreased ER stress levels in AGS and Ncl-N87, decreased expression levels of PERK, ATF4, SLC7A11, GPX4 (Fig. S6A), and elevated levels of LPO (Fig. S7A), Fe^2+^ (Fig. S8A, B), and ROS (Fig. S10A, B) content, and overexpression of YY1 reversed these changes. Similarly, overexpression of ACSL3 caused HGC-27 and MKN45 to show results opposite to those described above, and knockdown of YY1 also reversed these changes (Fig. S6B, S7B, S8C, D, S10C, D). It has been demonstrated that YY1 promotes USP37 transcription, and when ACSL3 and YY1 were knocked down or overexpressed, the changes in USP37 expression appeared to be consistent with those of PERK, ATF4, SLC7A11, and GPX4 (Fig. S6A, B). These results collectively suggest that ACSL3 suppresses ferroptosis in GC by activating the PERK pathway via YY1. Furthermore, USP37 may be involved in how ACSL3 regulates the downstream PERK-EIF2α-ATF4-SLC7A11 axis.

### USP37 enhanced deubiquitination of PERK in GC cells, modulating the UPR and GC progression

**Fig. 5:**
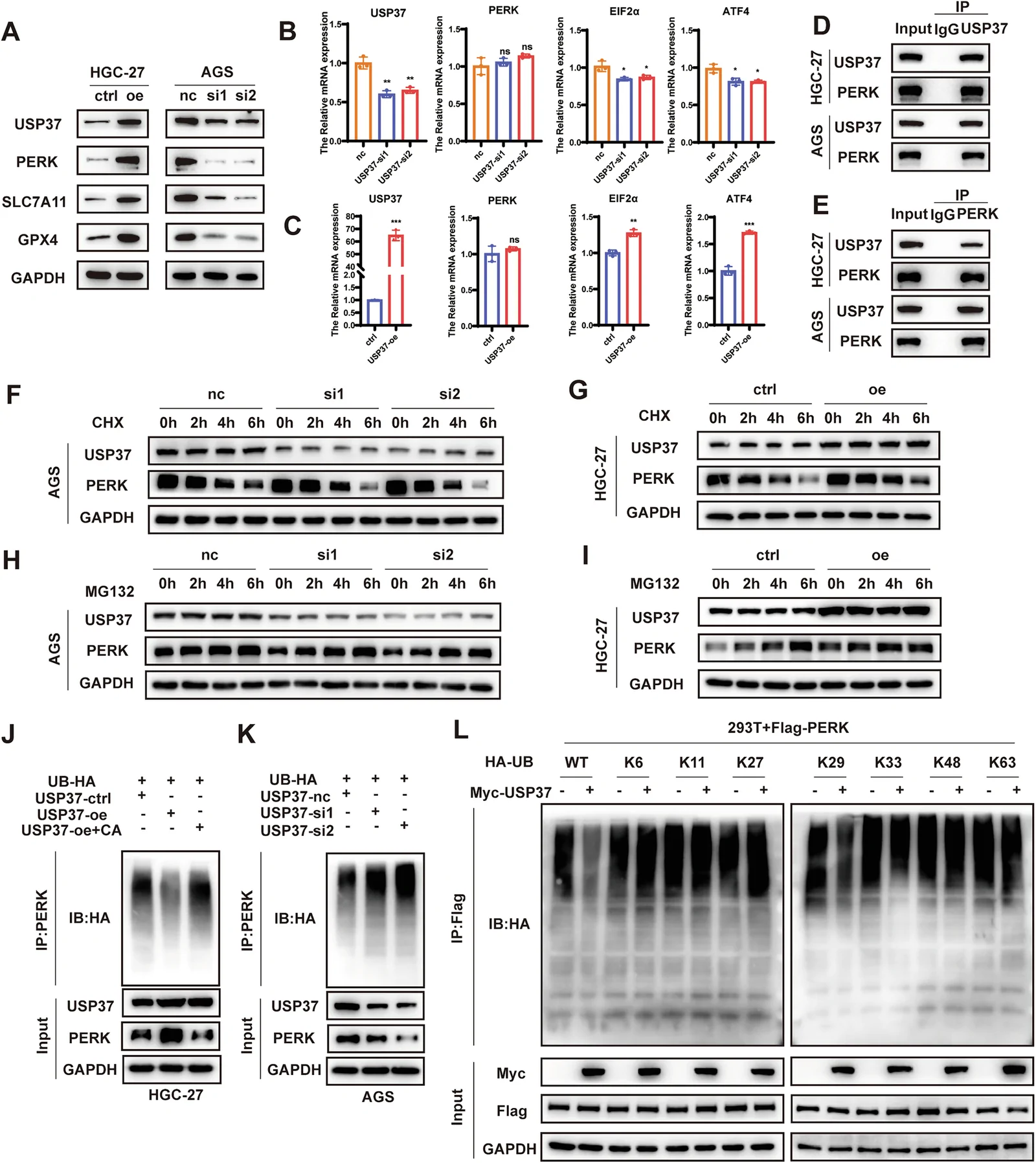
USP37 enhanced deubiquitination of PERK in GC cells, modulating the UPR and GC progression. **A** Western blot detection of USP37, PERK, SLC7A11 and GPX4 in HGC-27 transfected with USP37 or vector control and in AGS transfected with siUSP37 RNA or siRNA control. **B** RT-qPCR analyzed the mRNA of USP37, PERK, EIF2α and ATF4 in AGS transfected with siUSP37 RNA or siRNA control. **C** RT-qPCR analyzed the mRNA of USP37, PERK, EIF2α, and ATF4 in HGC-27 transfected with USP37 or vector control. **D**, **E** Co-IP assays revealed the interaction between USP37 and PERK in HGC-27 and AGS. **F** AGS cells transfected with siUSP37 RNA or siRNA control were treated with CHX for various lengths of time. Western blot assay was used to detect USP37 and PERK protein levels. **G** HGC-27 cells transfected with USP37 or vector control were treated with CHX for various lengths of time. Western blot assay was used to detect USP37 and PERK protein levels. **H** AGS cells transfected with siUSP37 RNA or siRNA control were treated with MG132 for various lengths of time. Western blot assay was used to detect USP37 and PERK protein levels. **I** HGC-27 cells transfected with USP37 or vector control were treated with MG132 for various lengths of time. Western blot assay was used to detect USP37 and PERK protein levels. **J** Anti-HA immunoblotting assay of PERK polyubiquitination in HGC-27 transfected with USP37, vector control or enzyme-dead mutant USP37-C350A (CA) after overexpression. **K** Anti-HA immunoblotting assay of PERK polyubiquitination in AGS transfected with siUSP37 RNA or siRNA control. **L** HA-WT, K6, K11, K27, K29, K33, K48 or K63 Ub were co-transfected with Flag-PERK and Myc-USP37 into HEK293T cells. After treatment with 10 μM MG132 for 6 h, cell lysates were subjected to ubiquitination assay and the ubiquitination level of PERK detected by HA antibody. Data are represented as the mean ± SD. Statistical analysis was performed using Student’s t-test, *p < 0.05; **p < 0.01; ***p < 0.001.

USP37 is a member of the USP deubiquitinating enzyme family and often acts to stabilize proteins through its deubiquitinating function [28, 29]. To verify the relationship between USP37 and PERK, overexpression of USP37 was found to increase the expression levels of PERK and SLC7A11, GPX4 in HGC-27 (Fig. 5A) and MKN45 (Fig. S14A), and knockdown of USP37 resulted in reduced levels of PERK and SLC7A11, GPX4 expression in AGS (Fig. 5A) and Ncl-N87 (Fig. S14A). At the mRNA level, knockdown or overexpression of USP37 could not affect the altered mRNA levels of PERK, but was able to cause mRNA levels of genes downstream of the PERK pathway (Fig. 5B). To verify the potential protein interactions between USP37 and PERK, Co-IP results showed that USP37 had protein interactions with PERK (Fig. 5D, E; Fig S14B, C). Subsequently, stimulation of GC cells with knockdown and overexpression of USP37 using 10 mM Cycloheximide (CHX) revealed that the rate of PERK degradation was significantly accelerated after knockdown of USP37 (Fig. 5F; Fig. S14D) and slowed down after overexpression of USP37 (Fig. 5G; Fig. S14E). To further verify how USP37 affects PERK protein levels, GC cells were stimulated with 10 mM MG132. While low-expression of USP37 reduced the protein level of PERK, MG132 was able to reverse this down-regulation, and overexpression of USP37 elevated the protein level of PERK, but MG132 also reversed this up-regulation (Fig. 5H, I; Fig. S14F, G). Overexpression of USP37 decreased the ubiquitination level of PERK in HGC-27 (Fig. 5J) and MKN45 (Fig. S14H), which is counteracted by the enzyme-dead mutant USP37-C350A (CA), and knockdown of USP37 showed the opposite result in AGS (Fig. 5K) and Ncl-N87 (Fig. S14I). These results suggested that USP37 relies on the proteasome pathway to inhibit the degradation of PERK. There are different processes of protein ubiquitination, including K6-, K11-, K27-, K29-, K33-, K48- and K63-linked ubiquitination. To elucidate the specific ubiquitin linkage regulated by USP37 in PERK deubiquitination, comprehensive ubiquitination assays were conducted in HEK-293T cells co-transfected with Flag-PERK and various HA-tagged ubiquitin constructs, including wild-type Ub, K6-, K11-, K27-, K29-, K33-, K48-, and K63-linked Ub, along with either Myc-USP37 expression vector or empty vector. Our results demonstrated that USP37 specifically reduced K29-linked ubiquitination, indicating that K29 linkage is crucial for USP37-mediated deubiquitination and subsequent stabilization of PERK (Fig. 5L). Knockdown of YY1 in GC cells revealed decreased expression levels of PERK, ATF4, SLC7A11, and GPX4 (Fig. S6C), and increased levels of LPO (Fig. S7C), Fe^2+^ (Fig. S9A, B), and ROS (Fig. S11A, B), and overexpression of USP37 was able to reverse these changes. Similarly, after overexpression of YY1 in GC cells revealed increased expression levels of PERK, ATF4, SLC7A11, and GPX4 (Fig. S6D), and decreased levels of LPO (Fig. S7D), Fe^2+^ (Fig. S9C, D), and ROS (Fig. S11C, D), and knockdown of USP37 also reversed these changes. Knockdown of ACSL3 in GC cells revealed decreased expression levels of ATF4, SLC7A11, and GPX4 (Fig. S6E), and increased levels of LPO (Fig. S7E), Fe^2+^ (Fig. S12A, B), and ROS (Fig. S13A, B), and overexpression of PERK was able to reverse these changes. Similarly, after overexpression of ACSL3 in GC cells revealed increased expression levels of ATF4, SLC7A11, and GPX4 (Fig. S6F), and decreased levels of LPO (Fig. S7F), Fe^2+^ (Fig. S12C, D), and ROS (Fig. S13C, D), and knockdown of PERK also reversed these changes. These results suggest that ACSL3 promotes the YY1-USP37-PERK axis, thereby inhibiting ferroptosis in GC.

### The ACSL3-YY1-USP37-PERK signaling axis modulated GC progression through ferroptosis regulation in vivo

**Fig. 6:**
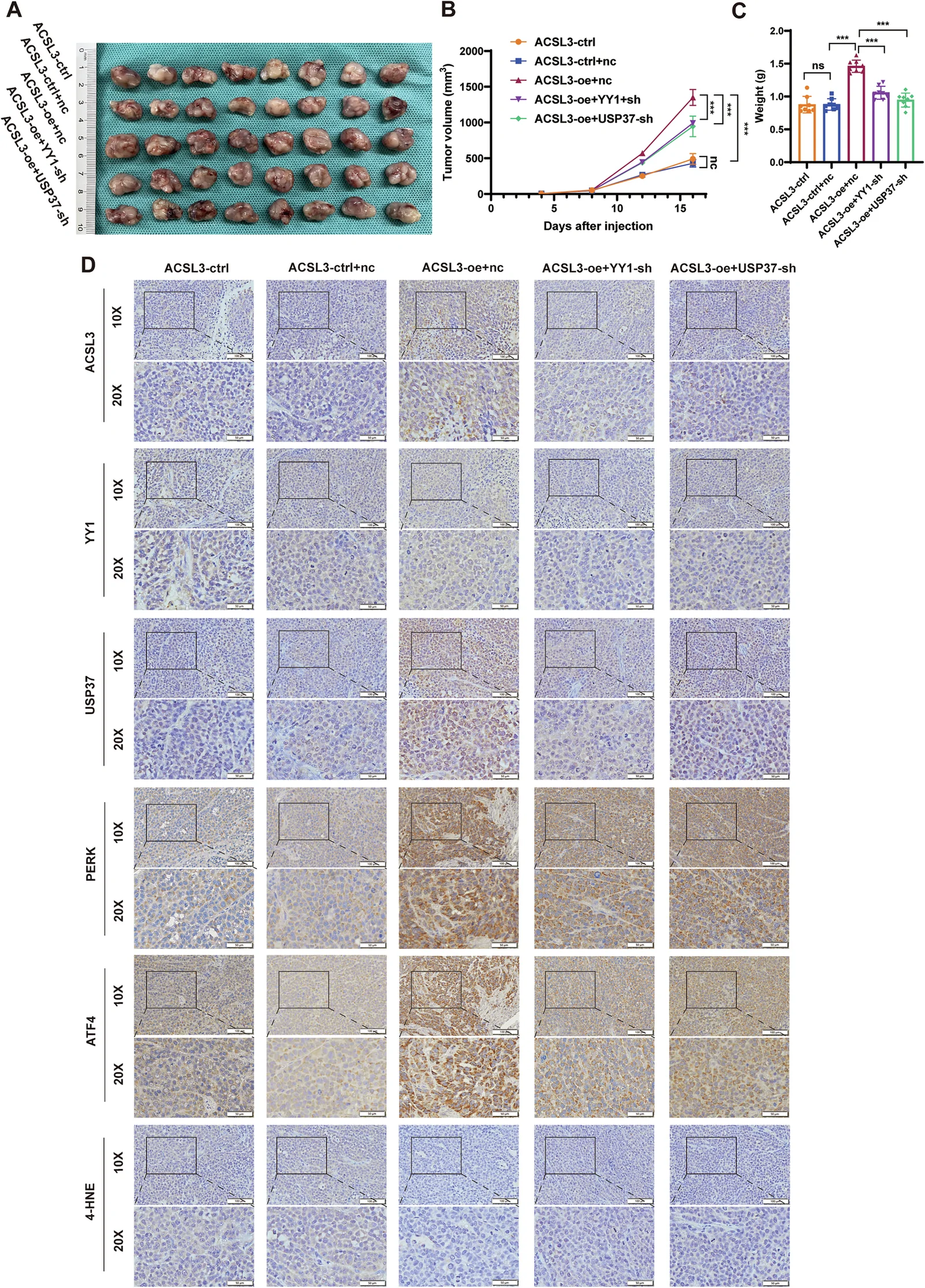
The ACSL3-YY1-USP37-PERK signaling axis modulated GC progression through ferroptosis regulation in vivo. **A–C** Nude mice were subcutaneously injected with stable-transfer cell lines HGC-27 (ACSL3-ctrl, ACSL3-ctrl+nc, ACSL3-oe+nc, ACSL3-oe+YY1-sh, ACSL3-oe+USP37-sh), and the tumors were removed after 16 days. **A** General view of tumor mass of each indicated group at 16 days after cell injection. **B** Tumor size was measured with vernier calipers, and tumor volume was calculated every 4 days after inoculation, and plotted the growth curves of each group of tumors. **C** Tumor weights of the groups at the end of the experiment. **D** Representative IHC images of the expressions of ACSL3, YY1, USP37, PERK, ATF4 and 4-HNE protein in the aforementioned xenograft tumors. Data are represented as the mean ± SD. Statistical analysis was performed using Student’s t-test, *p < 0.05; **p < 0.01; ***p < 0.001.

To test whether ACSL3 also regulates GC growth via YY1, USP37 in vivo, the HGC-27 cell line was used to construct stable-transfer cell lines (ACSL3-ctrl, ACSL3-ctrl+nc, ACSL3-oe+nc, ACSL3-oe+YY1-sh, ACSL3-oe+USP37-sh) to establish a subcutaneous tumor model by inoculating axillae of BALB/c nude mice respectively. The 4-week-old nude mice were randomly divided into 5 groups (*n* = 8) and collected 16 days after injection (Fig. 6A), tumor volumes were measured every 4 days (Fig. 6B) and measured the tumors weight (Fig. 6C). The expression levels of ACSL3, YY1, USP37, PERK, ATF4, 4-HNE were analyzed by IHC in subcutaneous tumor tissues of each group (Fig. 6D). High expression of ACSL3 significantly down-regulated the level of ferroptosis and promoted the growth of tumors, whereas further knocking down of YY1 or USP37 down-regulated the tumor growth-promoting effect of ACSL3. The Ncl-N87 cell line was also used to construct stable-transfer cell lines (ACSL3-nc, ACSL3-nc+ctrl, ACSL3-sh+ctrl, ACSL3-sh+YY1-oe, ACSL3-sh+USP37-oe) to establish a subcutaneous tumor model (Fig. S15A–C), and the expression levels of ACSL3, YY1, USP37, PERK, ATF4, 4-HNE were also analyzed by IHC (Fig. S15D). The HGC-27 cell line was used to construct stable-transfer cell lines (ACSL3-ctrl, ACSL3-oe, ACSL3-oe+PERK-sh) to establish a subcutaneous tumor model (Fig. S16A–C), and Ncl-N87 cell line was also used to construct stable-transfer cell lines (ACSL3-nc, ACSL3-sh, ACSL3-sh+PERK-oe) to establish a subcutaneous tumor model (Fig. S16D–F). Similarly, the expression levels of ACSL3, PERK, ATF4, 4-HNE were also analyzed by IHC (Fig. S16G, H). Similarly, knockdown of PERK suppressed the tumor growth-promoting effect of ACSL3. The in vivo results confirmed that low ACSL3 expression significantly promoted ferroptosis and inhibited tumor growth. Conversely, the tumor-promoting effect of ACSL3 was enhanced by the subsequent overexpression of YY1, USP37, or PERK. In summary, ACSL3 suppresses ferroptosis and promotes GC progression through the regulation of the ACSL3-YY1-USP37-PERK signaling axis. This axis represents a potential therapeutic target for the treatment of GC.

### ACSL3 was positively correlated with USP37 and PERK expression in GC patients

**Fig. 7:**
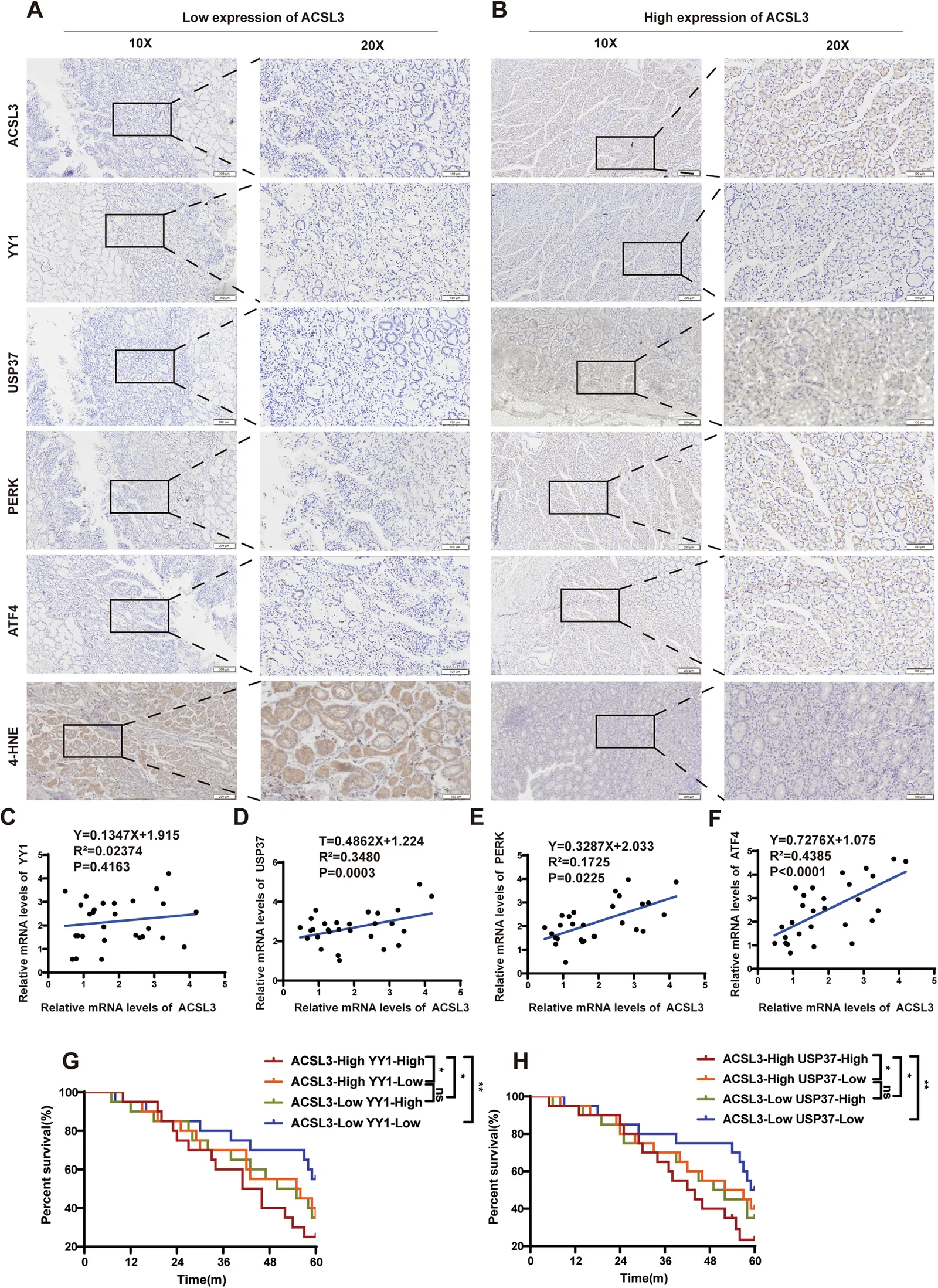
ACSL3 was positively correlated with USP37 and PERK expression in GC patients. **A** GC tissues from patients were assayed for ACSL3 expression. Representative immunohistochemical images of ACSL3, YY1, USP37, PERK, ATF4, and 4-HNE protein expression in patients with low ACSL3 expression. **B** Representative immunohistochemical images of ACSL3, YY1, USP37, PERK, ATF4, and 4-HNE protein expression in patients with high ACSL3 expression. **C** RT-qPCR analyzed the mRNA of ACLS3 and YY1 of GC tissues (*n* = 30), and a correlation graph was plotted between ACSL3 and YY1. **D** RT-qPCR analyzed the mRNA of ACLS3 and USP37 of GC tissues (*n* = 30), and a correlation graph was plotted between ACSL3 and USP37. **E** RT-qPCR analyzed the mRNA of ACLS3 and PERK of GC tissues (*n* = 30), and a correlation graph was plotted between ACSL3 and PERK. **F** RT-qPCR analyzed the mRNA of ACLS3 and ATF4 of GC tissues (*n* = 30), and a correlation graph was plotted between ACSL3 and ATF4. **G** GC tissues from patients were assayed for ACSL3, YY1, and USP37 expression. Kaplan–Meier curves of overall survival were performed on GC patients in different groups (high ACSL3 expression and high YY1 expression, high ACSL3 expression and low YY1 expression, low ACSL3 expression and high YY1 expression, low ACSL3 expression and low YY1 expression). **H** Kaplan–Meier curves of overall survival were performed on GC patients in different groups (high ACSL3 expression and high USP37 expression, high ACSL3 expression and low USP37 expression, low ACSL3 expression and high USP37 expression, low ACSL3 expression and low USP37 expression). Data are represented as the mean ± SD. Statistical analysis was performed using Student’s t-test, *p < 0.05; **p < 0.01; ***p < 0.001.

To validate the consistency of results in clinical samples, GC tissues from patients with GC for ACSL3 expression measurement and determined the expression levels of ACSL3, YY1, USP37, PERK, ATF4, 4-HNE were determined by IHC. The results showed that the expression of ACSL3-USP37-PERK-ATF4 was positively correlated with each other, and it significantly inhibited ferroptosis (Fig. 7A, B). Further, by determining the mRNA levels in tumor tissues, ACSL3 did not correlate significantly with the expression of YY1 (Fig. 7C), whereas it correlated significantly and positively with the expression of USP37, PERK, and ATF4 (Fig. 7D, E, F). The effect of this expression correlation on the prognosis of GC patients was investigated. High expression of ACSL3 was associated with significantly poorer prognosis compared to low expression, whereas high ACSL3 expression accompanied by low YY1 or USP37 expression correlated with improved prognosis (Fig. 7G, H). The results showed that ACSL3 inhibited ferroptosis through YY1 and USP37 and thus correlated with the prognosis of GC patients

**Fig. 8:**
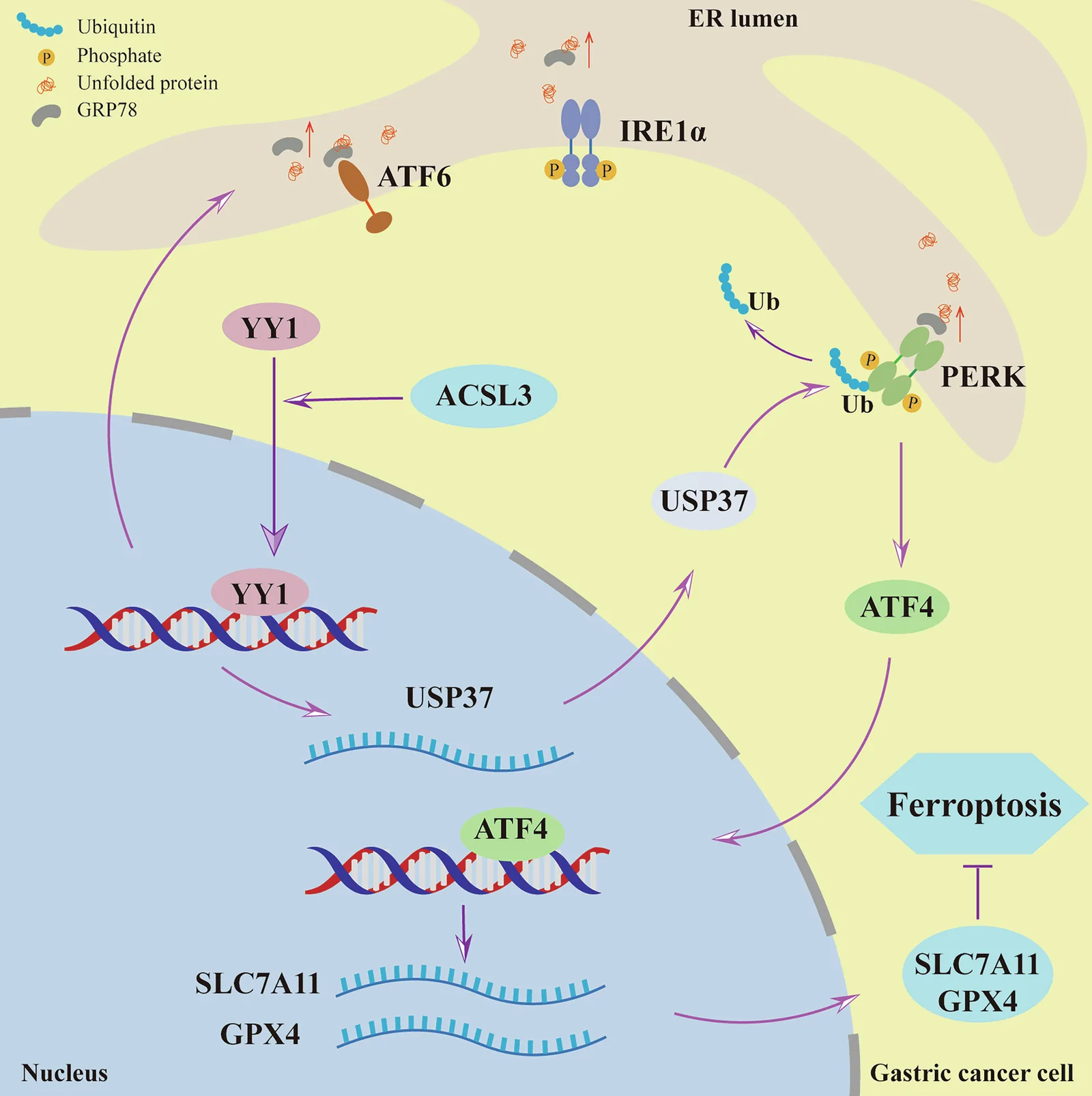
The role of ACSL3-YY1-USP37-PERK regulatory axis in the inhibition of ferroptosis in GC. ACSL3 mediated the nuclear translocation of YY1, thereby initiating ER stress and the UPR (Fig. 8). Concurrently, YY1 interacted with the promoter region of USP37, thereby enhancing its transcriptional expression. USP37, in turn, facilitated the deubiquitination of PERK, culminating in the inhibition of ferroptosis in GC.

In light of the presented findings, we introduced a novel model delineating the ACSL3-YY1-USP37-PERK signaling axis as a pivotal regulator of ferroptosis in GC. ACSL3 mediated the nuclear translocation of YY1, thereby initiating ER stress and the UPR (Fig. 8). Concurrently, YY1 enhanced the transcriptional expression of USP37 by interacting with its promoter region. USP37, in turn, inhibited ferroptosis in GC by facilitating the deubiquitination of PERK.

## Discussion

Due to the heterogeneity and complexity of GC, GC is considered to be an aggressive malignant tumor. Moreover, most patients with advanced GC often present with a poor prognosis [30, 31]. However, the efficacy of tumor molecular targeted therapy for GC is still limited and unstable [32, 33]. Therefore, it is essential to find and identify a specific and efficient molecular target for early diagnosis and targeted therapy of GC.

In normal tissues, only liver cells and adipocytes have the de novo FA synthesis pathway, but tumor cells are metabolically reprogrammed to have the ability to synthesize de novo FAs themselves [6, 34]. Cancer cell membranes are characterized by increased MUFA side chain to saturated fatty acid acyl (SFA) side chain ratio and MUFA to polyunsaturated fatty acid acyl (PUFA) side chain ratio, which reduces lipotoxicity and sensitivity to ferroptosis [7]. A xenograft model in nude mice was established, and intraperitoneally injected with OA and L-OHP. The results showed that OA promoted tumor progression and reduced tumor sensitivity to L-OHP. Combined with previous literature reports, MUFA was confirmed to promote the progression of GC. The ACSL family is a key enzyme in MUFA metabolism. Most of the previous studies on ACSL and GC have focused on ACSL4. In this thesis, we focused on the important role played by ACSL3 in GC progression.

Analysis of clinical samples and analyzing clinical information revealed significantly elevated ACSL3 expression in GC tissues, which correlated with chemotherapy resistance and poor prognosis. The results are similar to the conclusions of previous studies on ACSL3 and other tumors [35–37]. Suggesting that ACSL3 may be highly expressed in tumors and associated with poor prognosis of patients, and may be used as an effective tumor marker and target for targeted therapy.

To further investigate the mechanism of ACSL3 inhibiting ferroptosis in GC cells, differential expression analysis of ACSL3-regulated downstream genes was performed using the TCGA database, identifying USP37 as the most significantly dysregulated candidate for further study. Ubiquitin-specific proteases (USPs), as a subclass of deubiquitinating enzymes, play key roles in protein deubiquitination [38]. Ubiquitination is a common form of protein post-translational modification (PTM) that involves an ATP-dependent process of attaching ubiquitin to specific proteins and inducing protein degradation [39]. Similar to other PTMS, ubiquitination is a reversible process. Deubiquitinating enzymes (DUBs) are peptidases that reverse ubiquitination by separating protein substrates from counterclaims, a phenomenon known as deubiquitination [40]. The USP family is the largest and most diverse family of DUBs, characterized by the presence of a conserved catalytic domain called the USP domain. In addition, various USPs also possess other domains or motifs, such as ubiquitin-like domains and zinc finger ubiquitin-binding domain, which further enhance the function of USP [41]. USP plays an important role in many physiological processes such as protein degradation, DNA repair, chromatin remodeling, and cell cycle regulation [42]. However, there is no evidence that USP37 expression is directly regulated by ACSL family or affected by FA levels. Therefore, we hypothesized that ACSL3 may indirectly affect USP37 expression in some way.

By using Human TFDB, GTRD, and PROMO databases to predict the potential upstream TFs of USP37, and YY1 was finally selected as the TF of USP37 due to its most direct and obvious role. These 9 genes that were publicly owned by the 3 databases were knocked down in AGS, and it was found that the mRNA level of USP37 was significantly decreased after knocking down YY1, TBP, and POU2F1, but knockdown of TBP and POU2F1 only reduced USP37 mRNA levels but did not affect its protein expression. This is related to multiple factors such as post-transcriptional regulation and feedback control mechanisms. During the conversion of mRNA into protein, various regulatory processes may occur, including splicing, modification, stability, transport, and degradation. Certain proteins can also alter their own expression through diverse feedback control mechanisms. Any changes in these processes may lead to inconsistencies between the transcript and protein levels.

YY1 is a zinc finger protein that is highly conserved in all vertebrates and plays a dual role in regulating gene transcription due to the presence of an N-terminal activation domain and a C-terminal repression domain [43, 44]. YY1 can regulate transcriptional regulation, epigenetic regulation, cell proliferation and other important cell physiological functions by exerting the functions of traditional DNA-binding factors, or binding with non-coding RNA and protein [45, 46]. The expression level of YY1 is often increased in many tumors, such as colorectal cancer [47], breast cancer [48], cervical cancer [49, 50] and liver cancer [51], which is associated with drug resistance, recurrence, metastasis, and poor prognosis. The specific mechanism by which ACSL3 promotes YY1 nuclear transport remains unclear in this study. Western blot and IF experiments revealed that ACSL3 promotes YY1 nuclear translocation. In addition, YY1 can undergo nuclear translocation and change its function as a TF under certain special stimuli and influences [25, 52, 53]. Furthermore, previous studies have demonstrated that nuclear translocation of molecules involves multiple steps and molecular interactions. For example, EGF–EGFR signaling directly phosphorylates TSPAN8 at Ser129 via the kinase AKT, thereby orchestrates TSPAN8 nuclear translocation. ERK1/2 regulates the phosphorylation of RFNG at the S255 site, thereby promoting RFNG nuclear translocation and mediating L-OHP resistance. These findings provide potential avenues for further investigation into the pathogenic mechanisms of YY1 in GC. While we need to explore these possibilities further, current experimental limitations prevent us from definitively determining the specific regulatory mechanism. Our study found that ACSL3 can induce nuclear translocation of YY1 and enhance the ER stress mediated by YY1. Further studies showed that changes in PERK expression in the UPR pathway can significantly affect the expression of SLC7A11 and GPX4, suggesting that PERK may be a downstream gene regulated by ACSL3.

The deubiquitinating enzyme USP37 was initially identified as a potent regulator of the cell cycle, capable of accelerating G1/S conversion [54, 55]. Recent studies have shown that USP37 also plays an important role in the occurrence and development of tumors. USP37 is abnormally overexpressed in breast cancer tissues and promotes the stemness and invasion ability of breast cancer cells [56]. USP37 regulates the stability of HIF-2α and is associated with the loss of tumor suppressor von Hippel–Lindau gene in clear cell renal cell carcinoma [57]. Our results showed that USP37 induced deubiquitination of PERK through the ubiquitin site of K29, which further promoted the expression of ATF4, SLC7A11, and GPX4, and inhibited the ferroptosis of GC cells.

However, this study identified the ACSL3-YY1-USP37-PERK regulatory axis, which promotes GC progression by inhibiting ferroptosis. Unfortunately, effective targeted drugs such as ACSL3-specific inhibitors or USP37 enzyme activity site-specific blockers remain unavailable. USP37 enzyme site-specific blockers to further investigate the inhibitory effects of exogenous drugs on these key molecules in vivo.

Overall, the present study elucidated a previously unrecognized ACSL3-YY1-USP37-PERK regulatory axis that orchestrated ferroptosis suppression and facilitated GC progression. These findings not only provide mechanistic insights into the molecular regulation of ferroptosis in GC but also establish ACSL3 as a promising diagnostic biomarker and therapeutic target for GC intervention.

## Supplementary information

Supplementary materials

Supplementary Table 1

Supplementary Table 2

Supplementary Table 3

Supplementary Table 4

Original data of Western Blots

## Data Availability

The datasets generated and/or analyzed during this study are available from the corresponding author upon reasonable request.
