# Supplementary material for: ACSL3 inhibits ferroptosis in gastric cancer through the activation of unfolded protein response following endoplasmic reticulum stress
Source: Cell Death Dis. 2026 Jul 7;17(1):844. doi: 10.1038/s41419-026-09068-3 (PMC13623976; doi:10.1038/s41419-026-09068-3)
Supplement: Supplementary file 1 [file 41419_2026_9068_MOESM1_ESM.docx]

**Supplementary materials**

**Supplementary Figure 1. ACSL3 inhibited ferroptosis in GC cells. (A-C)** OA significantly promoted GC tumor progression and reduced L-OHP sensitivity in the xenograft mouse model. Ncl-N87 cells were injected subcutaneously in the left axilla of BALB/c nude mice aged 4 weeks (n = 4, randomly assigned). Tumor size was measured with vernier calipers and tumor volume was calculated every 4 days after inoculation, oleic acid (OA, 2 mg/kg, intratumoral), oxaliplatin (L-OHP, 5 mg/kg, intratumor injection), or PBS control was injected on days 6, 8, 12, and 14 after inoculation, and tumors were collected and weighed on day 16. **(A)** Tumor size in each group at the end of the experiment. **(B)** Tumor growth delay of GC cells treated with PBS control, L-OHP treatment, oleic acid administration and OA plus L-OHP combination. **(C)** Tumor weights of the groups at the end of the experiment. **(D)** Western blot assay of ACSL3 expression in MKN45 transfected with ACSL3 or vector control and in Ncl-N87 transfected with shACSL3 RNA or shRNA control. **(E)** Western blot detection of ACSL3, SLC7A11 and GPX4 expression levels in the groups of GC cells, ACSL3-ctrl, ACSL3-oe, ACSL3-nc, ACSL3-sh1, ACSL3-sh2. **(F)** Cell flow assay detection of lipid peroxides levels in the groups of ACSL3-ctrl, ACSL3-oe in MKN45. **(G)** Detection of MDA levels in the groups of ACSL3-ctrl, ACSL3-oe in MKN45. **(H)** Cell flow assay detection of lipid peroxides levels in the groups of ACSL3-nc, ACSL3-sh1, ACSL3-sh2 in Ncl-N87. **(I)** Detection of MDA levels in the groups of ACSL3-nc, ACSL3-sh1, ACSL3-sh2 in Ncl-N87. **(J)** Detection of ROS levels in the groups of ACSL3-ctrl, ACSL3-oe in MKN45. **(K)** Detection of ROS levels in the groups of ACSL3-nc, ACSL3-sh1, ACSL3-sh2 in Ncl-N87. **(L)** Detection of Fe^2+^ levels in the groups of ACSL3-ctrl, ACSL3-oe in MKN45. **(M)** Detection of Fe^2+^ levels in the groups of ACSL3-nc, ACSL3-sh1, ACSL3-sh2 in Ncl-N87. Data are represented as the mean ± SD. Statistical analysis was performed using Student’s t-test, *p < 0.05; **p < 0.01; ***p < 0.001.

**Supplementary Figure 1. ACSL3 inhibited ferroptosis in GC cells.** **(A)** Detection of ROS levels in the groups of ACSL3-nc, ACSL3-sh1, ACSL3-sh2, ACSL3-nc+ ferrostatin‑1, ACSL3-sh1+ ferrostatin‑1, ACSL3-sh2+ ferrostatin‑1 in AGS. **(B)** Detection of Fe^2+^ levels in the groups of ACSL3-nc, ACSL3-sh1, ACSL3-sh2, ACSL3-nc+ferrostatin‑1, ACSL3-sh1+ferrostatin‑1, ACSL3-sh2+ferrostatin‑1 in AGS. **(C)** Detection of ROS levels in the groups of ACSL3-nc, ACSL3-sh1, ACSL3-sh2, ACSL3-nc+ferrostatin‑1, ACSL3-sh1+ferrostatin‑1, ACSL3-sh2+ferrostatin‑1 in Ncl-N87. **(D)** Detection of Fe^2+^ levels in the groups of ACSL3-nc, ACSL3-sh1, ACSL3-sh2, ACSL3-nc+ferrostatin‑1, ACSL3-sh1+ferrostatin‑1, ACSL3-sh2+ferrostatin‑1 in Ncl-N87. **(E)** Cell flow assay detection of lipid peroxides levels in the groups of ACSL3-nc, ACSL3-sh1, ACSL3-sh2, ACSL3-nc+ferrostatin‑1, ACSL3-sh1+ferrostatin‑1, ACSL3-sh2+ferrostatin‑1 in AGS. **(F)** Detection of MDA levels in the groups of ACSL3-nc, ACSL3-sh1, ACSL3-sh2, ACSL3-nc+ferrostatin‑1, ACSL3-sh1+ferrostatin‑1, ACSL3-sh2+ferrostatin‑1 in AGS. **(G)** Cell flow assay detection of lipid peroxides levels in the groups of ACSL3-nc, ACSL3-sh1, ACSL3-sh2, ACSL3-nc+ferrostatin‑1, ACSL3-sh1+ferrostatin‑1, ACSL3-sh2+ferrostatin‑1 in Ncl-N87. **(H)** Detection of MDA levels in the groups of ACSL3-nc, ACSL3-sh1, ACSL3-sh2, ACSL3-nc+ferrostatin‑1, ACSL3-sh1+ferrostatin‑1, ACSL3-sh2+ferrostatin‑1 in Ncl-N87. Data are represented as the mean ± SD. Statistical analysis was performed using Student’s t-test, *p < 0.05; **p < 0.01; ***p < 0.001.

**Supplementary Figure 3. ACSL3 inhibited ferroptosis in GC cells. (A)** Detection of ROS levels in the groups of ACSL3-nc, ACSL3-sh1, ACSL3-sh2, ACSL3-nc+Erastin, ACSL3-sh1+Erastin, ACSL3-sh2+Erastin in AGS. **(B)** Detection of Fe^2+^ levels in the groups of ACSL3-nc, ACSL3-sh1, ACSL3-sh2, ACSL3-nc+Erastin, ACSL3-sh1+Erastin, ACSL3-sh2+Erastin in AGS. **(C)** Detection of ROS levels in the groups of ACSL3-nc, ACSL3-sh1, ACSL3-sh2, ACSL3-nc+Erastin, ACSL3-sh1+Erastin, ACSL3-sh2+Erastin in Ncl-N87. **(D)** Detection of Fe^2+^ levels in the groups of ACSL3-nc, ACSL3-sh1, ACSL3-sh2, ACSL3-nc+Erastin, ACSL3-sh1+Erastin, ACSL3-sh2+Erastin in Ncl-N87. **(E)** Cell flow assay detection of lipid peroxides levels in the groups of ACSL3-nc, ACSL3-sh1, ACSL3-sh2, ACSL3-nc+Erastin, ACSL3-sh1+Erastin, ACSL3-sh2+Erastin in AGS. **(F)** Detection of MDA levels in the groups of ACSL3-nc, ACSL3-sh1, ACSL3-sh2, ACSL3-nc+Erastin, ACSL3-sh1+Erastin, ACSL3-sh2+Erastin in AGS. **(G)** Cell flow assay detection of lipid peroxides levels in the groups of ACSL3-nc, ACSL3-sh1, ACSL3-sh2, ACSL3-nc+Erastin, ACSL3-sh1+Erastin, ACSL3-sh2+Erastin in Ncl-N87. **(H)** Detection of MDA levels in the groups of ACSL3-nc, ACSL3-sh1, ACSL3-sh2, ACSL3-nc+Erastin, ACSL3-sh1+Erastin, ACSL3-sh2+Erastin in Ncl-N87. Data are represented as the mean ± SD. Statistical analysis was performed using Student’s t-test, *p < 0.05; **p < 0.01; ***p < 0.001.

**Supplementary Figure 4. ACSL3 inhibited ferroptosis in GC cells. (A)** Detection of ROS levels in the groups of ACSL3-ctrl, ACSL3-oe, ACSL3-ctrl+Erastin, ACSL3-oe+Erastin in HGC-27. **(B)** Detection of Fe^2+^ levels in the groups of ACSL3-ctrl, ACSL3-oe, ACSL3-ctrl+Erastin, ACSL3-oe+Erastin in HGC-27. **(C)** Detection of ROS levels in the groups of ACSL3-ctrl, ACSL3-oe, ACSL3-ctrl+Erastin, ACSL3-oe+Erastin in MKN45. **(D)** Detection of Fe^2+^ levels in the groups of ACSL3-ctrl, ACSL3-oe, ACSL3-ctrl+Erastin, ACSL3-oe+Erastin in MKN45. **(E)** Cell flow assay detection of lipid peroxides levels in the groups of ACSL3-ctrl, ACSL3-oe, ACSL3-ctrl+Erastin, ACSL3-oe+Erastin in HGC-27. **(F)** Detection of MDA levels in the groups of ACSL3-ctrl, ACSL3-oe, ACSL3-ctrl+Erastin, ACSL3-oe+Erastin in HGC-27. **(G)** Cell flow assay detection of lipid peroxides levels in the groups of ACSL3-ctrl, ACSL3-oe, ACSL3-ctrl+Erastin, ACSL3-oe+Erastin in MKN45. **(H)** Detection of MDA levels in the groups of ACSL3-ctrl, ACSL3-oe, ACSL3-ctrl+Erastin, ACSL3-oe+Erastin in MKN45. Data are represented as the mean ± SD. Statistical analysis was performed using Student’s t-test, *p < 0.05; **p < 0.01; ***p < 0.001.

**Supplementary Figure 5. ACSL3-mediated regulation of YY1 nuclear translocation augments ER stress response and suppressed ferroptosis in GC cells. (A)** Western blot detection of ACSL3 and YY1 expression levels in the groups of GC cells, ACSL3-ctrl, ACSL3-oe, ACSL3-nc, ACSL3-sh1, ACSL3-sh2. **(B)** Western blot detection of ACSL3 and YY1 expression in the nucleus and the cytoplasm in Ncl-N87 transfected with shACSL3 RNA or shRNA control. **(C)** Representative images of immunofluorescence staining on YY1 (green) and DAPI (blue) in Ncl-N87 transfected with shACSL3 RNA or shRNA control. **(D)** Western blot detection of ER stress marker molecules in MKN45 transfected with YY1 or vector control and in Ncl-N87 transfected with siYY1 RNA or siRNA control. **(E)** Western blot detection of ER stress marker molecules in MKN45 transfected with ACSL3 or vector control and in Ncl-N87 transfected with shACSL3 RNA or shRNA control. **(F)** Western blot detection of UPR marker molecules in MKN45 transfected with ACSL3 or vector control and in Ncl-N87 transfected with shACSL3 RNA or shRNA control.

**Supplementary Figure 6. ACSL3 regulation of YY1 nuclear translocation promoted ER stress response and inhibited ferroptosis. (A)**Western blot detection of ACSL3, YY1, USP37, ER stress marker molecules, UPR marker molecules, ferroptosis marker molecules in AGS and Ncl-N87 (ACSL3-nc+YY1-ctrl, ACSL3-nc+YY1-oe, ACSL3-sh1+YY1- ctrl, ACSL3-sh1+YY1-oe, ACSL3-sh2+YY1-ctrl, ACSL3-sh2+YY1-oe). **(B)** Western blot detection of ACSL3, YY1, USP37, ER stress marker molecules, UPR marker molecules, ferroptosis marker molecules in HGC-27 and MKN45 (ACSL3-ctrl+YY1-nc, ACSL3-ctrl+YY1-sh1, ACSL3-ctrl+YY1-sh2, ACSL3-oe+YY1-nc, ACSL3-oe+YY1-sh1, ACSL3-oe+YY1-sh2). **(C)** Western blot detection of YY1, USP37, UPR marker molecules, ferroptosis marker molecules in AGS and Ncl-N87 (YY1-nc+USP37-ctrl, YY1-nc+USP37-oe, YY1-sh1+USP37-ctrl, YY1-sh1+USP37-oe, YY1-sh2+USP37-ctrl, YY1-sh2+USP37-oe). **(D)** Western blot detection of YY1, USP37, UPR marker molecules, ferroptosis marker molecules in HGC-27 and MKN45 (YY1-ctrl+USP37-nc, YY1-ctrl+USP37-sh1, YY1-ctrl+USP37-sh2, YY1-oe+USP37-nc, YY1-oe+USP37-sh1, YY1-oe+USP37-sh2). **(E)** Western blot detection of ACSL3, UPR marker molecules, ferroptosis marker molecules in AGS and Ncl-N87 (ACSL3-nc+PERK-ctrl, ACSL3-nc+PERK-oe, ACSL3-sh1+PERK-ctrl, ACSL3-sh1+PERK-oe, ACSL3-sh2+PERK-ctrl, ACSL3-sh2+ PERK-oe). **(F)** Western blot detection of ACSL3, UPR marker molecules, ferroptosis marker molecules in HGC-27 and MKN45 (ACSL3-ctrl+PERK-nc, ACSL3-ctrl+ PERK-sh1, ACSL3-ctrl+ PERK-sh2, ACSL3-oe+ PERK-ctrl, ACSL3-oe+ PERK-sh1, ACSL3-oe+ PERK-sh2). **Supplementary Figure 7. ACSL3 affected ferroptosis by affecting YY1, USP37 and PERK. (A)** Cell flow assay detection of lipid peroxides levels in the groups of ACSL3-nc+YY1-ctrl, ACSL3-nc+YY1-oe, ACSL3-sh1+YY1-ctrl, ACSL3-sh1+YY1-oe, ACSL3-sh2+YY1-ctrl, ACSL3-sh2+YY1-oe in AGS. **(B)** Cell flow assay detection of lipid peroxides levels in the groups of ACSL3-ctrl+YY1-nc, ACSL3-ctrl+YY1-sh1, ACSL3-ctrl+YY1-sh2, ACSL3-oe+YY1-nc, ACSL3-oe+YY1-sh1, ACSL3-oe+YY1-sh2 in HGC-27. **(C)** Cell flow assay detection of lipid peroxides levels in the groups of YY1-nc+USP37-ctrl, YY1-nc+USP37-oe, YY1-sh1+USP37-ctrl, YY1-sh1+USP37-oe, YY1-sh2+USP37-ctrl, YY1-sh2+USP37-oe in AGS. **(D)** Cell flow assay detection of lipid peroxides levels in the groups of YY1-ctrl+USP37-nc, YY1-ctrl+USP37-sh1, YY1-ctrl+USP37-sh2, YY1-oe+USP37-nc, YY1-oe+USP37-sh1, YY1-oe+USP37-sh2 in HGC-27. **(E)** Cell flow assay detection of lipid peroxides levels in the groups of ACSL3-nc+PERK-ctrl, ACSL3-nc+PERK-oe, ACSL3-sh1+ PERK-ctrl, ACSL3-sh1+PERK-oe, ACSL3-sh2+PERK-ctrl, ACSL3-sh2+PERK-oe in AGS. **(F)** Cell flow assay detection of lipid peroxides levels in the groups of ACSL3-ctrl+PERK-nc, ACSL3-ctrl+ PERK-sh1, ACSL3-ctrl+ PERK-sh2, ACSL3-oe+ PERK-ctrl, ACSL3-oe+ PERK-sh1, ACSL3-oe+ PERK-sh2 in HGC-27. Data are represented as the mean ± SD. Statistical analysis was performed using Student’s t-test, *p < 0.05; **p < 0.01; ***p < 0.001.

**Supplementary Figure 8. ACSL3 affected ferroptosis by affecting YY1. (A)** Detection of Fe^2+^ levels in the groups of ACSL3-nc+YY1-ctrl, ACSL3-nc+YY1-oe, ACSL3-sh1+YY1-ctrl, ACSL3-sh1+YY1-oe, ACSL3-sh2+YY1-ctrl, ACSL3-sh2+YY1-oe in AGS. **(B)** Detection of Fe^2+^ levels in the groups of ACSL3-nc+YY1-ctrl, ACSL3-nc+YY1-oe, ACSL3-sh1+YY1-ctrl, ACSL3-sh1+YY1-oe, ACSL3-sh2+YY1-ctrl, ACSL3-sh2+YY1-oe in Ncl-N87. **(C)** Detection of Fe^2+^ levels in the groups of ACSL3-ctrl+YY1-nc, ACSL3-ctrl+YY1-sh1, ACSL3-ctrl+YY1-sh2, ACSL3-oe+YY1-nc, ACSL3-oe+YY1-sh1, ACSL3-oe+YY1-sh2 in HGC-27. **(D)** Detection of Fe^2+^ levels in the groups of ACSL3-ctrl+YY1-nc, ACSL3-ctrl+YY1-sh1, ACSL3-ctrl+YY1-sh2, ACSL3-oe+YY1-nc, ACSL3-oe+YY1-sh1, ACSL3-oe+YY1-sh2 in MKN. Data are represented as the mean ± SD. Statistical analysis was performed using Student’s t-test, *p < 0.05; **p < 0.01; ***p < 0.001.

**Supplementary Figure 9. YY1 affected ferroptosis by affecting USP37. (A)** Detection of Fe^2+^ levels in the groups of YY1-nc+USP37-ctrl, YY1-nc+USP37-oe, YY1-sh1+USP37-ctrl, YY1-sh1+USP37-oe, YY1-sh2+USP37-ctrl, YY1-sh2+USP37-oe in AGS. **(B)** Detection of Fe^2+^ levels in the groups of YY1-nc+USP37-ctrl, YY1-nc+USP37-oe, YY1-sh1+USP37-ctrl, YY1-sh1+USP37-oe, YY1-sh2+USP37-ctrl, YY1-sh2+USP37-oe in Ncl-N87. **(C)** Detection of Fe^2+^ levels in the groups of YY1-ctrl+USP37-nc, YY1-ctrl+USP37-sh1, YY1-ctrl+USP37-sh2, YY1-oe+USP37-nc, YY1-oe+USP37-sh1, YY1-oe+USP37-sh2 in HGC-27. **(D)** Detection of Fe^2+^ levels in the groups of YY1-ctrl+USP37-nc, YY1-ctrl+USP37-sh1, YY1-ctrl+USP37-sh2, YY1-oe+USP37-nc, YY1-oe+USP37-sh1, YY1-oe+USP37-sh2 in MKN. Data are represented as the mean ± SD. Statistical analysis was performed using Student’s t-test, *p < 0.05; **p < 0.01; ***p < 0.001.

**Supplementary Figure 10. ACSL3 affected ferroptosis by affecting YY1. (A)** Detection of ROS levels in the groups of ACSL3-nc+YY1-ctrl, ACSL3-nc+YY1-oe, ACSL3-sh1+YY1-ctrl, ACSL3-sh1+YY1-oe, ACSL3-sh2+YY1-ctrl, ACSL3-sh2+YY1-oe in AGS. **(B)** Detection of ROS levels in the groups of ACSL3-nc+YY1-ctrl, ACSL3-nc+YY1-oe, ACSL3-sh1+YY1-ctrl, ACSL3-sh1+YY1-oe, ACSL3-sh2+YY1-ctrl, ACSL3-sh2+YY1-oe in Ncl-N87. **(C)** Detection of ROS levels in the groups of ACSL3-ctrl+YY1-nc, ACSL3-ctrl+YY1-sh1, ACSL3-ctrl+YY1-sh2, ACSL3-oe+YY1-nc, ACSL3-oe+YY1-sh1, ACSL3-oe+YY1-sh2 in HGC-27. **(D)** Detection of ROS levels in the groups of ACSL3-ctrl+YY1-nc, ACSL3-ctrl+YY1-sh1, ACSL3-ctrl+YY1-sh2, ACSL3-oe+YY1-nc, ACSL3-oe+YY1-sh1, ACSL3-oe+YY1-sh2 in MKN. Data are represented as the mean ± SD. Statistical analysis was performed using Student’s t-test, *p < 0.05; **p < 0.01; ***p < 0.01.

**Supplementary Figure 11. YY1 affected ferroptosis by affecting USP37. (A)** Detection of ROS levels in the groups of YY1-nc+USP37-ctrl, YY1-nc+USP37-oe, YY1-sh1+USP37-ctrl, YY1-sh1+USP37-oe, YY1-sh2+USP37-ctrl, YY1-sh2+USP37-oe in AGS. **(B)** Detection of ROS levels in the groups of YY1-nc+USP37-ctrl, YY1-nc+USP37-oe, YY1-sh1+USP37-ctrl, YY1-sh1+USP37-oe, YY1-sh2+USP37-ctrl, YY1-sh2+USP37-oe in Ncl-N87. **(C)** Detection of ROS levels in the groups of YY1-ctrl+USP37-nc, YY1-ctrl+USP37-sh1, YY1-ctrl+USP37-sh2, YY1-oe+USP37-nc, YY1-oe+USP37-sh1, YY1-oe+USP37-sh2 in HGC-27. **(D)** Detection of ROS levels in the groups of YY1-ctrl+USP37-nc, YY1-ctrl+USP37-sh1, YY1-ctrl+USP37-sh2, YY1-oe+USP37-nc, YY1-oe+USP37-sh1, YY1-oe+USP37-sh2 in MKN. Data are represented as the mean ± SD. Statistical analysis was performed using Student’s t-test, *p < 0.05; **p < 0.01; ***p < 0.01.

**Supplementary Figure 12. ACSL3 affected ferroptosis by affecting PERK. (A)** Detection of Fe^2+^ levels in the groups of ACSL3-nc+PERK-ctrl, ACSL3-nc+PERK-oe, ACSL3-sh1+PERK-ctrl, ACSL3-sh1+PERK-oe, ACSL3-sh2+PERK-ctrl, ACSL3-sh2+PERK-oe in AGS. **(B)** Detection of Fe^2+^ levels in the groups of ACSL3-nc+PERK-ctrl, ACSL3-nc+PERK-oe, ACSL3-sh1+PERK-ctrl, ACSL3-sh1+PERK-oe, ACSL3-sh2+PERK-ctrl, ACSL3-sh2+PERK-oe in Ncl-N87. **(C)** Detection of Fe^2+^ levels in the groups of ACSL3-con+PERK-nc, ACSL3-ctrl+PERK-sh1, ACSL3-ctrl+PERK-sh2, ACSL3-oe+PERK-nc, ACSL3-oe+PERK-sh1, ACSL3-oe+PERK-sh2 in HGC-27. **(D)** Detection of Fe^2+^ levels in the groups of ACSL3-ctrl+PERK-nc, ACSL3-ctrl+PERK-sh1, ACSL3-ctrl+PERK-sh2, ACSL3-oe+PERK-nc, ACSL3-oe+PERK-sh1, ACSL3-oe+PERK-sh2 in MKN. Data are represented as the mean ± SD. Statistical analysis was performed using Student’s t-test, *p < 0.05; **p < 0.01; ***p < 0.001.

**Supplementary Figure 13. ACSL3 affected ferroptosis by affecting PERK. (A)** Detection of ROS levels in the groups of ACSL3-nc+PERK-ctrl, ACSL3-nc+PERK-oe, ACSL3-sh1+PERK-ctrl, ACSL3-sh1+PERK-oe, ACSL3-sh2+PERK-ctrl, ACSL3-sh2+PERK-oe in AGS. **(B)** Detection of ROS levels in the groups of ACSL3-nc+PERK-ctrl, ACSL3-nc+PERK-oe, ACSL3-sh1+PERK-ctrl, ACSL3-sh1+PERK-oe, ACSL3-sh2+PERK-ctrl, ACSL3-sh2+PERK-oe in Ncl-N87. **(C)** Detection of ROS levels in the groups of ACSL3-con+PERK-nc, ACSL3-ctrl+PERK-sh1, ACSL3-ctrl+PERK-sh2, ACSL3-oe+PERK-nc, ACSL3-oe+PERK-sh1, ACSL3-oe+PERK-sh2 in HGC-27. **(D)** Detection of ROS levels in the groups of ACSL3-ctrl+PERK-nc, ACSL3-ctrl+PERK-sh1, ACSL3-ctrl+PERK-sh2, ACSL3-oe+PERK-nc, ACSL3-oe+PERK-sh1, ACSL3-oe+PERK-sh2 in MKN. Data are represented as the mean ± SD. Statistical analysis was performed using Student’s t-test, *p < 0.05; **p < 0.01; ***p < 0.001.

**Supplementary Figure 14. USP37 enhanced deubiquitination of PERK in GC cells, modulating the unfolded protein response and GC progression.** **(A)** Western blot detection of USP37, PERK, SLC7A11 and GPX4 in MKN45 transfected with USP37 or vector control and in Ncl-N87 transfected with siUSP37 RNA or siRNA control. **(B, C)** Co-IP assays revealed the interaction between USP37 and PERK in MKN45 and Ncl-N87. **(D)** Ncl-N87 cells transfected with siUSP37 RNA or siRNA control were treated with cycloheximide for various lengths of time. Western blot assay was used to detect USP37 and PERK protein levels. **(E)** MKN45 cells transfected with USP37 or vector control were treated with cycloheximide for various lengths of time. Western blot assay was used to detect USP37 and PERK protein levels. **(F)** Ncl-N87 cells transfected with siUSP37 RNA or siRNA control were treated with MG132 for various lengths of time. Western blot assay was used to detect USP37 and PERK protein levels. **(G)** MKN45 cells transfected with USP37 or vector control were treated with MG132 for various lengths of time. Western blot assay was used to detect USP37 and PERK protein levels. **(H)** Anti-HA immunoblotting assay of PERK polyubiquitination in MKN45 transfected with USP37, vector control or enzyme-dead mutant USP37-C350A (CA) after overexpression. **(I)** Anti-HA immunoblotting assay of PERK polyubiquitination in Ncl-N87 transfected with USP37, vector control or mutation at the enzyme activation site after overexpression.

**Supplementary Figure 15. The ACSL3-YY1-USP37-PERK signaling axis modulated GC progression through ferroptosis regulation *in vivo*. (A-C)** Nude mice were subcutaneously injected with stable-transfer cell lines Ncl-N87 (ACSL3-nc, ACSL3-nc+ctrl, ACSL3-sh+ctrl, ACSL3-sh+ YY1-oe, ACSL3-sh+ USP37-oe), and the tumors were removed after 16 days. **(A)** General view of tumor mass of each indicated group at 16 days after cell injection. **(B)** Tumor size was measured with vernier calipers and tumor volume was calculated every 4 days after inoculation, and plotted the growth curves of each group of tumors. **(C)** Tumor weights of the groups at the end of the experiment. **(D)** Representative immunohistochemistry images of the expressions of ACSL3, YY1, USP37, PERK, ATF4 and 4-HNE protein in the aforementioned xenograft tumors. Data are represented as the mean ± SD. Statistical analysis was performed using Student’s t-test, *p < 0.05; **p < 0.01; ***p < 0.001.

**Supplementary Figure 16. The ACSL3-YY1-USP37-PERK signaling axis modulated GC progression through ferroptosis regulation *in vivo*. (A-C)** Nude mice were subcutaneously injected with stable-transfer cell lines HGC-27 (ACSL3-ctrl, ACSL3-oe, ACSL3-oe+PERK-sh), and the tumors were removed after 16 days. **(A)** General view of tumor mass of each indicated group at 16 days after cell injection. **(B)** Tumor size was measured with vernier calipers and tumor volume was calculated every 4 days after inoculation, and plotted the growth curves of each group of tumors. **(C)** Tumor weights of the groups at the end of the experiment. **(D-F)** Nude mice were subcutaneously injected with stable-transfer cell lines Ncl-N87 (ACSL3-nc, ACSL3-sh, ACSL3-sh+PERK-oe), and the tumors were removed after 16 days. **(D)** General view of tumor mass of each indicated group at 16 days after cell injection. **(E)** Tumor size was measured with vernier calipers and tumor volume was calculated every 4 days after inoculation, and plotted the growth curves of each group of tumors. **(F)** Tumor weights of the groups at the end of the experiment. **(G)** Representative immunohistochemistry images of the expressions of ACSL3, PERK, ATF4 and 4-HNE expression in xenograft tumors derived from HGC-27 cells with stable transfection of ACSL3-ctrl, ACSL3-oe, or ACSL3-oe+PERK-sh. **(H)** Representative immunohistochemistry images of the expressions of ACSL3, PERK, ATF4 and 4-HNE expression in xenograft tumors derived from Ncl-N87 cells with stable transfection of ACSL3-nc, ACSL3-sh, ACSL3-sh+PERK-oe. Data are represented as the mean ± SD. Statistical analysis was performed using Student’s t-test, *p < 0.05; **p < 0.01; ***p < 0.001.
