# Supplementary material for: ACSL3 inhibits ferroptosis in gastric cancer through the activation of unfolded protein response following endoplasmic reticulum stress
Source: Cell Death Dis. 2026 Jul 7;17(1):844. doi: 10.1038/s41419-026-09068-3 (PMC13623976; doi:10.1038/s41419-026-09068-3)
Supplement: Supplementary file 2 [file 41419_2026_9068_MOESM2_ESM.docx]

**Supplementary Table 1.** The relationship between the ACSL3 expression in GC tissues and clinicopathologic features.

| **Clinicopathological features（N=200）** | **Low expression of ACSL3 N (%)** | **High expression of ACSL3 N (%)** | ***p* value** |
| --- | --- | --- | --- |
| Age | 62.28±8.864 | 59.95±11.061 | 0.105 |
| Gender |  |  |  |
| Male | 74（69.2） | 63（67.7） | 0.830 |
| Female | 33（30.8） | 30（32.3） |  |
| Tumor size |  |  |  |
| ≤5 cm | 73（68.2） | 39（41.9） | <0.001 |
| >5 cm | 34（31.8） | 54（58.1） |  |
| Pathological T stage |  |  |  |
| I-II | 51（47.7） | 30（32.3） | 0.027 |
| III-IV | 56（52.3） | 63（67.7） |  |
| Diferentiation degree |  |  |  |
| Low | 38（35.5） | 48（51.6） | 0.002 |
| Medium–high | 69（64.5） | 45（48.4） |  |

N=Number
