# Supplementary material for: ACSL3 inhibits ferroptosis in gastric cancer through the activation of unfolded protein response following endoplasmic reticulum stress
Source: Cell Death Dis. 2026 Jul 7;17(1):844. doi: 10.1038/s41419-026-09068-3 (PMC13623976; doi:10.1038/s41419-026-09068-3)
Supplement: Supplementary file 3 [file 41419_2026_9068_MOESM3_ESM.docx]

**Supplementary Table 2.**

Sequences of siRNAs.

| Name | Sequence (5' – 3)' |
| --- | --- |
| YY1-si1 | CUGAGGUAACUCUUCUUGCTT |
| YY1-si2 | GCAAGAAGAGUUACCUCAGTT |
| USP37-si1 | CAAAAGAGCUACCGAGUUA |
| USP37-si2 | GCAUACACUUGCCCUGUUA |
| TBP-si1 | UAUCCACAGUGAAUCUUGGTT |
| TBP-si2 | UCAAACCCAGAAUUGUUCUTT |
| POU2F1-si1 | GATCCGCAACTGGGAACCTGG |
| POU2F1-si2 | GCAGCGAGTCAAGATGAGAGT |

Sequences of shRNAs.

| Name | Primer Sequence (5’→3’) |
| --- | --- |
| ACSL3-sh1 | CCGGGCCTTCAAGCTGAAACGCAAACTCGAGTTTGCGTTTCAGCTTGAAGGCTTTTTG |
| ACSL3-sh2 | CCGGGCGGACATTGAGCGAATGTATCTCGAGATACATTCGCTCAATGTCCGCTTTTTG |
| YY1-sh1 | CGGCTTCGAGGATCAGATTCT |
| YY1-sh2 | CCTCCTGATTATTCAGAATAT |
| USP37-sh1 | CGGAGTGGCTACATCTTCTTT |
| USP37-sh2 | CCGGATTTGCAGAAGATGATA |
