# Supplementary material for: ACSL3 inhibits ferroptosis in gastric cancer through the activation of unfolded protein response following endoplasmic reticulum stress
Source: Cell Death Dis. 2026 Jul 7;17(1):844. doi: 10.1038/s41419-026-09068-3 (PMC13623976; doi:10.1038/s41419-026-09068-3)
Supplement: Supplementary file 4 [file 41419_2026_9068_MOESM4_ESM.docx]

**Supplementary Table 3.** Primer sequences for RT-qPCR.

| Name | Primer Sequence (5’→3’) |
| --- | --- |
| ACSL3-F | GGCGTAGCGGTTTTGACA |
| ACSL3-R | CCAGTCCTTCCCAACAACGA |
| USP37-F | GAGTGCCCCTTTCCTCTCAC |
| USP37-R | AAGCAAGTGTGCAAAGCGTC |
| PERK-F | AGAGGTCTCGCTCCTTGAGT |
| PERK-R | GGCAAAGGGCTATGGGAGTT |
| EIF2A-F | GTTTTATGCCTGCCAAAGCGA |
| EIF2A-R | TCCCACACTTCCATTTGTCCC |
| ATF4-F | CTTGATGTCCCCCTTCGACC |
| ATF4-R | CTTGTCGCTGGAGAACCCAT |
| GFM1-F | TTGGGCACTTCCTGAGGGAT |
| GFM1-R | ACATCCTCCATCATGTCGGC |
| AGFG1-F | AAAGTGCTTCGACTGCGACC |
| AGFG1-R | TTTCACCCTGTGTGGTGGATT |
| SPTY2D1-F | TGTGTCCAAAAAGGCACCCT |
| SPTY2D1-R | ACTGTCCGCCTAGAGGGATT |
| NUS1-F | ATTAGCGTCTACGACCACCAA |
| NUS1-R | CAGCACCTTCACTGCCAAATG |
| UHMK1-F | AGAGAAACCATGGGCAGAAG |
| UHMK1-R | CAAGCCATGAAACAGCATCT |
| CLTC-F | ACGGTTGCTCTTGTTACGGA |
| CLTC-R | TGCATAGCTCCCACCACACG |
| SLC39A9-F | CATGCTGGCTTAGAGCGGAAT |
| SLC39A9-R | TGGTGCTGCCAATGCAAAGA |
| IREB2-F | AAGTCCATCCTGCTTGTCCG |
| IREB2-R | AAGTCCATCCTGCTTGTCCG |
| HMGCS1-F | GGCTATAAAGCTGCGGAGGG |
| HMGCS1-R | GCATGGTGAAAGAGCCAAAGG |
| COPB2-F | GTGGGGACAAGCCATACCTC |
| COPB2-R | GTGCTCTCAAGCCGGTAGG |
| MAPK6-F | CGGAAGACTTGGTGCTGAAGAT |
| MAPK6-R | TACGCTGAGAAGCTCCTGACGAT |
| DNAJC3-F | AGCAAGATTACAGAGAGGTC |
| DNAJC3-R | TGAGACTGTGCTTCCTTTTC |
| PPP1R15B-F | TGAGGATTGGGATGAGGAAG |
| PPP1R15B-R | TCTGGCAGCAGTCTGAATTG |
| TMED10-F | CATGAAGCATGGAGTAGAG |
| TMED10-R | CTCATTAGTGTCCCTCATC |
| TFRC-F | GTTGCAGCCTTACTATACGCC |
| TFRC-R | GCAGCACGTCGCTTATATTGG |
| COPB1-F | CCAATATGAAATGCCTGACTCC |
| COPB1-R | ATCTGGTCCCTGGTGAATTG |
| CUL3-F | TGTCGAATCTGAGCAAAGGC |
| CUL3-R | CATCCATGGTCATCGGAAAGG |
| ATP6V1A-F | GAGATCCTGTACTTCGCACTGG |
| ATP6V1A-R | GGGGATGTAGATGCTTTGGGT |
| USP32-F | GGCTGCTCGTGATATGCTGTTC |
| USP32-R | GTTTCTGGGCTGACACCTTGC |
| GAPDH-F | GCACCGTCAAGGCTGAGAAC |
| GAPDH-R | TGGTGAAGACGCCAGTGGA |
