# Supplementary material for: ACSL3 inhibits ferroptosis in gastric cancer through the activation of unfolded protein response following endoplasmic reticulum stress
Source: Cell Death Dis. 2026 Jul 7;17(1):844. doi: 10.1038/s41419-026-09068-3 (PMC13623976; doi:10.1038/s41419-026-09068-3)
Supplement: Supplementary file 5 [file 41419_2026_9068_MOESM5_ESM.docx]

**Supplementary Table 4. Primary antibodies used for western blotting (WB) and immunohistochemistry (IHC)**

| Antibody | Supplier | Catalog No. | Application |
| --- | --- | --- | --- |
| ACSL3 | Abcam | ab151959 | WB 1:1000  IHC 1:200 |
| GAPDH | Proteintech Group | 10494-1-AP | WB 1:1000 |
| SLC7A11 | ABclonal Biotechnology | A2413 | WB 1:1000 |
| GPX4 | Proteintech Group | 30388-1-AP | WB 1:1000 |
| USP37 | Proteintech Group | 18465-1-AP | WB 1:1000  IHC 1:200 |
| YY1 | Proteintech Group | 66281-1-Ig | WB 1:1000  IHC 1:200 |
| TBP | Proteintech Group | 22006-1-AP | WB 1:1000 |
| POU2F1 | Proteintech Group | 10387-1-AP | WB 1:1000 |
| PCNA | Proteintech Group | 60097-1-Ig | WB 1:1000 |
| CHOP | Proteintech Group | 15204-1-AP | WB 1:1000 |
| GRP78 | Proteintech Group | 11587-1-AP | WB 1:1000 |
| ERP5 | Proteintech Group | 18233-1-AP | WB 1:1000 |
| PERK | Cell Signaling Technology | 5683 | WB 1:1000  IHC 1:200 |
| IRE1a | Cell Signaling Technology | 3294 | WB 1:1000 |
| ATF6 | Cell Signaling Technology | 65880 | WB 1:1000 |
| ATF4 | Cell Signaling Technology | 11815 | WB 1:1000  IHC 1:200 |
| HA | Proteintech Group | 51064-2-AP | WB 1:10000 |
| Flag | Proteintech Group | 80801-2-RR | WB 1:10000 |
| Myc | Proteintech Group | 60003-2-Ig | WB 1:10000 |
