# Supplementary material for: ACSL3 inhibits ferroptosis in gastric cancer through the activation of unfolded protein response following endoplasmic reticulum stress
Source: Cell Death Dis. 2026 Jul 7;17(1):844. doi: 10.1038/s41419-026-09068-3 (PMC13623976; doi:10.1038/s41419-026-09068-3)
Supplement: Supplementary file 6 [file 41419_2026_9068_MOESM6_ESM.docx]

Figure 1

ACSL3 70 kDa

GAPDH 35 kDa

ACSL3 70 kDa

GAPDH 35 kDa

Figure 2

ACSL3 70 kDa

GAPDH 35 kDa

ACSL3 70 kDa

GAPDH 35 kDa

ACSL3 70 kDa

GAPDH 35 kDa

ACSL3 70 kDa

SLC7A11 55 kDa

GPX4 15 kDa

GAPDH 35 kDa

ACSL3 70 kDa

SLC7A11 55 kDa

GPX4 15 kDa

GAPDH 35 kDa

Figure 3

ACSL3 70 kDa

USP37 100 kDa

GAPDH 35 kDa

ACSL3 70 kDa

USP37 100 kDa

GAPDH 35 kDa

ACSL3 70 kDa

USP37 100 kDa

GAPDH 35 kDa

ACSL3 70 kDa

USP37 100 kDa

GAPDH 35 kDa

YY1 50 kDa

USP37 100 kDa

GAPDH 35 kDa

TBP 35 kDa

USP37 100 kDa

GAPDH 35 kDa

POU2F1 70 kDa

USP37 100 kDa

GAPDH 35 kDa

Figure 4

ACSL3 70 kDa

YY1 55 kDa

GAPDH 35 kDa

ACSL3 70 kDa

YY1 55 kDa

GAPDH 35 kDa

ACSL3 70 kDa

YY1 55 kDa

GAPDH 35 kDa

PCNA 35 kDa

YY1 55 kDa

CHOP 25 kDa

GRP78 55 kDa

ERP5 40 kDa

GAPDH 35 kDa

YY1 55 kDa

CHOP 25 kDa

GRP78 55 kDa

ERP5 40 kDa

GAPDH 35 kDa

ACSL3 70 kDa

CHOP 25 kDa

GRP78 55 kDa

ERP5 40 kDa

GAPDH 35 kDa

ACSL3 70 kDa

CHOP 25 kDa

GRP78 55 kDa

ERP5 40 kDa

GAPDH 35 kDa

ACSL3

70 kDa

CHOP 25 kDa

GRP78 55 kDa

ERP5 40 kDa

GAPDH 35 kDa

ACSL3 70 kDa

CHOP 25 kDa

GRP78 55 kDa

ERP5 40 kDa

GAPDH 35 kDa

ACSL3 70 kDa

PERK 130 kDa

IRE1a 110 kDa

ATF6 70 kDa

GAPDH 35 kDa

ACSL3 70 kDa

PERK 130 kDa

IRE1a 110 kDa

ATF6 70 kDa

GAPDH 35 kDa

PERK 130 kDa

SLC7A11 55 kDa

GPX4 15 kDa

GAPDH 35 kDa

IRE1a 110 kDa

SLC7A11 55 kDa

GPX4 15 kDa

GAPDH 35 kDa

ATF6 70 kDa

SLC7A11 55 kDa

GPX4 15 kDa

GAPDH35 kDa

Figure 5

USP37130 kDa

PERK 130 kDa

SLC7A11 55 kDa

GPX4 15 kDa

GAPDH35 kDa

USP37110 kDa

PERK 130 kDa

SLC7A1155 kDa

GPX4 15 kDa

GAPDH35 kDa

USP37130 kDa

PERK130 kDa

USP37130 kDa

PERK130 kDa

USP37130 kDa

PERK130 kDa

USP37130 kDa

PERK130 kDa

HA

USP37130 kDa

PERK130 kDa

GAPDH35 kDa

HA

USP37130 kDa

PERK130 kDa

GAPDH 35 kDa

USP37130 kDa

PERK130 kDa

GAPDH35 kDa

USP37130 kDa

PERK130 kDa

GAPDH55 kDa

USP37130 kDa

PERK130 kDa

GAPDH55 kDa

USP37130 kDa

PERK130 kDa

GAPDH35 kDa

HA

Myc 130 kDa

Flag 130 kDa

GAPDH35 kDa

HA

Myc 130 kDa

Flag 130 kDa

GAPDH 35 kDa

Figure S1

ACSL370 kDa

GAPDH35 kDa

ACSL370 kDa

GAPDH35 kDa

ACSL370 kDa

SLC7A1155 kDa

GPX415 kDa

GAPDH35 kDa

ACSL370 kDa

SLC7A1155 kDa

GPX415 kDa

GAPDH35 kDa

Figure S5

ACSL370 kDa

YY155 kDa

GAPDH35 kDa

ACSL370 kDa

YY155 kDa

GAPDH35 kDa

ACSL370 kDa

YY155 kDa

GAPDH35 kDa

PCNA35 kDa

YY155 kDa

CHOP25 kDa

GRP7870 kDa

ERP540 kDa

GAPDH35 kDa

YY155 kDa

CHOP25 kDa

GRP7870 kDa

ERP540 kDa

GAPDH35 kDa

ACSL370 kDa

CHOP25 kDa

GRP7870 kDa

ERP540 kDa

GAPDH35 kDa

ACSL370 kDa

CHOP25 kDa

GRP7870 kDa

ERP540 kDa

GAPDH35 kDa

ACSL370 kDa

PERK130 kDa

IRE1a110 kDa

ATF670 kDa

GAPDH35 kDa

ACSL370 kDa

PERK130 kDa

IRE1a130 kDa

ATF670 kDa

GAPDH35 kDa

Figure S6

ACSL370 kDa

YY155 kDa

USP37130 kDa

PERK130 kDa

ATF440 kDa

GRP7870 kDa

ERP540 kDa

CHOP25 kDa

SLC7A1155 kDa

GPX415 kDa

GAPDH35 kDa

ACSL370 kDa

YY155 kDa

USP37130 kDa

PERK130 kDa

ATF440 kDa

GRP7870 kDa

ERP540 kDa

CHOP25 kDa

SLC7A1155 kDa

GPX415 kDa

GAPDH35 kDa

ACSL370 kDa

YY155 kDa

USP37130 kDa

PERK130 kDa

ATF440 kDa

GRP7870 kDa

ERP540 kDa

CHOP25 kDa

SLC7A1155 kDa

GPX415 kDa

GAPDH35 kDa

ACSL370 kDa

YY155 kDa

USP37130 kDa

PERK130 kDa

ATF440 kDa

GRP7870 kDa

ERP540 kDa

CHOP25 kDa

SLC7A1155 kDa

GPX415 kDa

GAPDH35 kDa

YY155 kDa

USP37130 kDa

PERK130 kDa

ATF440 kDa

SLC7A1155 kDa

GPX415 kDa

GAPDH35 kDa

YY155 kDa

USP37130 kDa

PERK130 kDa

ATF440 kDa

SLC7A1155 kDa

GPX415 kDa

GAPDH35 kDa

YY155 kDa

USP37130 kDa

PERK130 kDa

ATF440 kDa

SLC7A1155 kDa

GPX415 kDa

GAPDH35 kDa

YY155 kDa

USP37130 kDa

PERK130 kDa

ATF440 kDa

SLC7A1155 kDa

GPX415 kDa

GAPDH35 kDa

ACSL370 kDa

PERK 130 kDa

ATF440 kDa

SLC7A1155 kDa

GPX415 kDa

GAPDH35 kDa

ACSL370 kDa

PERK130 kDa

ATF440 kDa

SLC7A1155 kDa

GPX415 kDa

GAPDH35 kDa

ACSL370 kDa

PERK130 kDa

ATF440 kDa

SLC7A1155 kDa

GPX4 15 kDa

GAPDH35 kDa

ACSL370 kDa

PERK130 kDa

ATF440 kDa

SLC7A1155 kDa

GPX415 kDa

GAPDH35 kDa

Figure S14

USP37110 kDa

PERK130 kDa

SLC7A11 55 kDa

GPX4 15 kDa

GAPDH35 kDa

USP37110 kDa

PERK 130 kDa

SLC7A1155 kDa

GPX4 15 kDa

GAPDH35 kDa

USP37100 kDa

PERK130 kDa

USP37100 kDa

PERK130 kDa

USP37100 kDa

PERK130 kDa

USP37100 kDa

PERK130 kDa

HA

USP37130 kDa

PERK130 kDa

GAPDH35 kDa

HA

USP37130 kDa

PERK130 kDa

GAPDH35 kDa

USP37130 kDa

PERK130 kDa

GAPDH35 kDa

USP37130 kDa

PERK130 kDa

GAPDH35 kDa

USP37130 kDa

PERK130 kDa

GAPDH35 kDa

USP37130 kDa

PERK130 kDa

GAPDH35 kDa
